# Recent Advances in Stereoselective Chemical *O*-Glycosylation Reactions

**DOI:** 10.3389/fmolb.2022.896187

**Published:** 2022-06-14

**Authors:** Mana Mohan Mukherjee, Rina Ghosh, John A. Hanover

**Affiliations:** ^1^ Laboratory of Cell and Molecular Biology, NIDDK, National Institutes of Health, Bethesda, MD, United States; ^2^ Department of Chemistry, Jadavpur University, Kolkata, India

**Keywords:** chemical synthesis, O-glycosylation, catalytic reaction, stereoselective techniques, 2-deoxy glycoside, reports of last decade

## Abstract

Carbohydrates involving glycoconjugates play a pivotal role in many life processes. Better understanding toward glycobiological events including the structure–function relationship of these biomolecules and for diagnostic and therapeutic purposes including tailor-made vaccine development and synthesis of structurally well-defined oligosaccharides (OS) become important. Efficient chemical glycosylation in high yield and stereoselectivity is however challenging and depends on the fine tuning of a protection profile to get matching glycosyl donor–acceptor reactivity along with proper use of other important external factors like catalyst, solvent, temperature, activator, and additive. So far, many glycosylation methods have been reported including several reviews also. In the present review, we will concentrate our discussion on the recent trend on α- and β-selective glycosylation reactions reported during the past decade.

## 1 Introduction

Glycoconjugates and polysaccharides (PS) are natural biopolymers, ubiquitous in nature, playing immense importance in several life processes like cell–cell communication and adhesion, embryogenesis, protein folding, blood group specificity, bacterial and viral infection, consequential immune responses, and cancer metastasis (Bleil and Wassarman, 1990; Giannis, 1994; Fukuda, 1995; Ernst et al., 2000; Lowe, 2001; Okajima and Irvine, 2002; Fuster and Esko, 2005). Whereas toward detail biological studies and bio-applications like epitope mapping (Shaw et al., 2002; Trujillo et al., 2004) enzyme characterization, bacteria-related tailor-made vaccine development (Ada and Isaacs, 2003; Kováč, 2006; Micoli et al., 2018; Kay et al., 2019; Micoli et al., 2019; Mettu et al., 2020) and also material synthesis (de Wit et al., 1993; Lichtenthaler, 2007) and isolation of carbohydrate-based biopolymers or oligomers in the pure homogeneous state in an appreciable yield is necessary but unfortunately it is difficult. Those oligomers or their modified analogs can be synthesized in pure state utilizing chemical or enzymatic glycosylation reactions. Owing to the difficulty in commercial availability and accessibility of some specific enzymes, especially in case of large-scale synthesis of oligosaccharide, chemical synthesis is a more practical pathway to get these molecules as per requirement. But, unlike peptides or nucleic acids, chemical synthesis of oligosaccharide (OS)/PS remains challenging due to more structural diversity and crucial handling of monomer protection–deprotection profiles for achieving appropriate matching reactivity of glycosyl donor and acceptor pair with the additional possibility of generation of anomeric mixtures. Glycosylation is the key reaction for OS/PS synthesis. Since the first report of Arthur glycosylation (Michael, 1879) followed by Fischer glycosylation (Fischer, 1891) there has been tremendous progress in this basic reaction, particularly during the past several decades (Boeckman et al., 2003; Ishiwata et al., 2010; Nigudkar and Demchenko, 2015; Mensink and Boltje, 2017; Adero et al., 2018; Bennett and Galan, 2018; Kulkarni et al., 2018; Nielsen and Pedersen, 2018; Panza et al., 2018; Yang et al., 2018; Ling and Bennett, 2019; Mukherjee et al., 2020; Rai and Kulkarni, 2020; Qin and Ye, 2021).

Despite a history of ∼142 years of glycosylation reactions, there is still no universal glycosylation method applied, in which a wide variety of any OS/PS can be synthesized. Although there are some elegant methodologies and chemo-selective techniques for synthesis of some groups of oligosaccharides in other situations, the synthesis of sugar oligomers become tricky. A little change in the reactivity of the glycosyl donor–acceptor partners based on their protection profile may sometimes lead to either formation of the desired glycoside only in low yield or even failing of a particular glycosylation method due to the formation of the undesired side product/s as the predominant one (Christensen et al., 2015).

The outcome of a glycosylation reaction broadly depends on the structures of glycosyl donors, acceptors, conformational constraints, promoters, additives and solvent (Kafle et al., 2016), and also on temperature (Chatterjee et al., 2018; Seeberger et al., 2022). For reducing the gap between a logically planned stereoselective chemical glycosylation and its corresponding actual outcome, a clear idea about the relative reactivity of glycosyl donors (L. Douglas et al., 1998; Zhang et al., 1999), acceptors (van der Vorm et al., 2017, 2018, 2019;Leng et al., 2018), activator (particularly with respect to its counter-anion) along with the state of the art of the glycosylation mechanism is necessary. A better understanding of the glycosylation pathways has become possible now owing to the recent elaborative mechanistic studies involving kinetic methods based on NMR, measurements of kinetic isotope effect, cation clock methods, and improved computational methods for ion pairs (Ayala et al., 2003; Crich and Chandrasekera, 2004; Huang et al., 2012a; Huang et al., 2012b; Adero et al., 2015; Frihed et al., 2015; Martin et al., 2016; Adero et al., 2018; Leng et al., 2018; Hansen et al., 2019; Lebedel et al., 2019; Santana et al., 2020; Crich, 2021; Fu et al., 2021).

**SCHEME 1:**
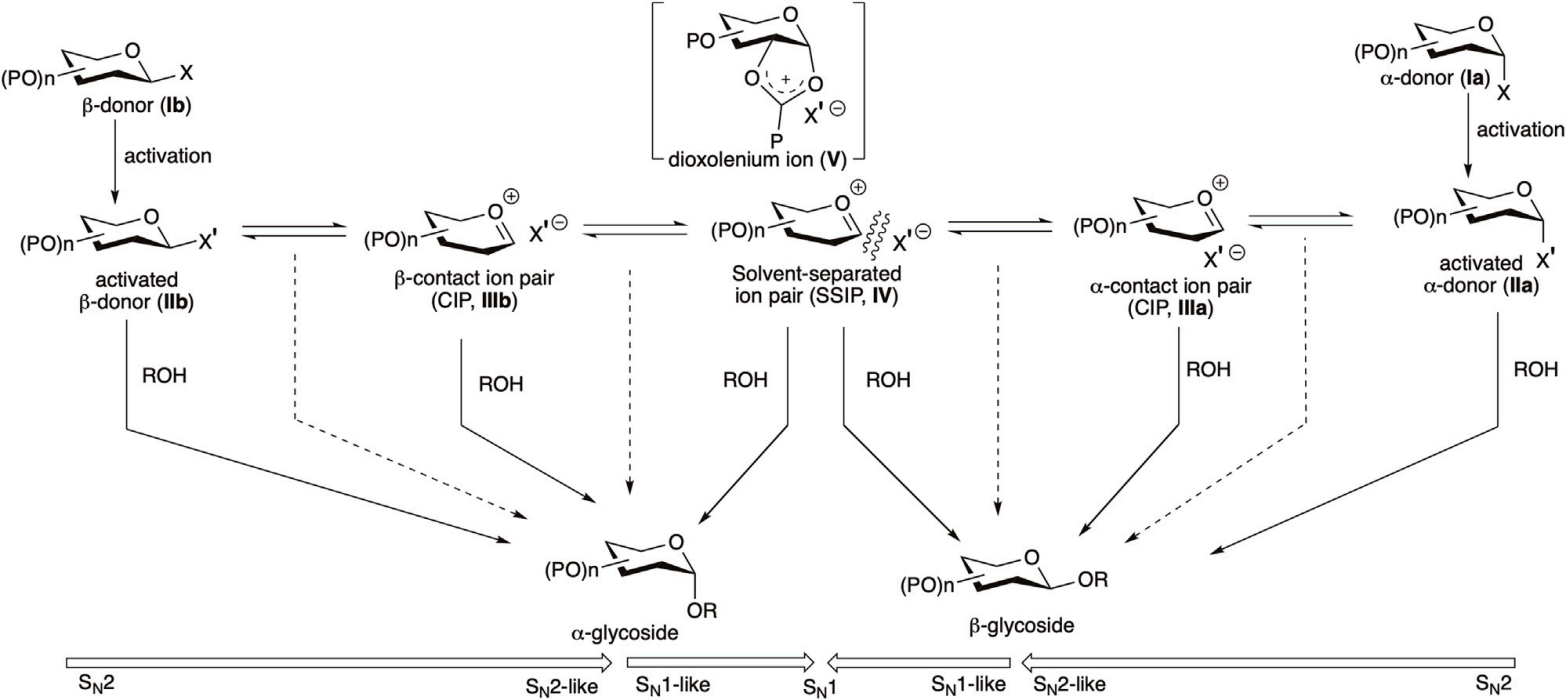
Outline of the general mechanism for chemical glycosylation.

Several of these advocate using bimolecular pathways, and others for the oxocarbenium intimate the ion pair-based unimolecular pathway (Huang et al., 2012a; 2012b; Adero et al., 2015; 2018). Glycosyl donors (**Ia/Ib**) are activated with a suitable activator, generating their corresponding activated donors (**IIa/IIb**, sometimes detectable by NMR spectroscopy) (Crich and Sun, 1997; Garcia and Gin, 2000; Liu and Gin, 2002; Walvoort et al., 2009; Kaeothip et al., 2012; Frihed et al., 2013; 2015). Following this, a fast anomarized α–β-equilibrium of the activated donor is possible where each anomer itself can react with the nucleophilic glycosyl acceptor or *via* some intermediate/s in the S_N_1–S_N_2 continuum (Scheme 1). In most of the cases of weak nucleophilic glycosyl acceptors, α-anomeric selectivity is observed, which is indicative of the leading role played by the activated β-glycosyl donor (**IIb**) or the corresponding intimate or contact ion pair (CIP) intermediate (**IIIb**) (Adero et al., 2018; Santana et al., 2020; Crich, 2021). 1,2-*Trans* linkages can be installed relatively more reliably using a neighboring group participation approach, where a participating group (such as *O*-acyl function) is present at C2–O, the reactive intermediates are intramolecularly trapped to provide a relatively stable acyloxonium ion (**V**). Here, attack by the nucleophilic glycosyl acceptor takes place stereoselectively from the opposite side of the ring, ultimately generating the 1,2-*trans* glycoside. But there is no general solution for the construction of 1,2-*cis* linkages. The situation is more complex in the absence of acyl protection on C2, and in such cases, involvement of a clear cut S_N_1 or S_N_2 pathway is much debated; there, either of the activated donors **IIa**/**IIb** or any one of or a series of electrophilic reactive intermediates (CIPs, **IIIa**/**IIIb**−SSIP, **IV**) can represent the product-forming intermediate (Scheme 1) (Crich, 2021), and the latter (SSIP, **IV**) may lead to decreased anomeric selectivity.

So far, very few generalized glycosylation reactions are possible toward the formation of the glycosides in high anomeric selectivity. Like, synthesis of 1,2-*trans* glycosides through anchimeric assistance by the C2–O–acyl group (Goodman, 1967; Fife et al., 1996; Nukada et al., 1998), Crich’s pre-activation based β-mannosylation of 4,6-*O*-benzylidenated mannosyl donors *via in situ* generation of mannosyl triflate (Crich and Sun, 1996, 1998b, a) and 4,6-*O*-di-tert-butylsilylene directed α-galactosylation with galactosyl donors (Imamura et al., 2003, 2006). But many other reported methods are having their own merits and demerits too. Recently, during the past 10 years, apart from glycosyl donor reactivity based glycosylation reactions, there has been a dramatic change in the glycosylation methodologies. Some reviews have been published recently focusing on separately different aspects of such glycosylation techniques (Nigudkar and Demchenko, 2015; Adero et al., 2018; Bennett and Galan, 2018; Kulkarni et al., 2018; Leng et al., 2018; Nielsen and Pedersen, 2018; Panza et al., 2018; Pohl, 2018; Ling and Bennett, 2019; van der Vorm et al., 2019; Yao et al., 2019; Khanam et al., 2020). Although by application of some modern expeditious glycosylation strategies like iterative one-pot glycosylation reactions (Huang et al., 2004; Biao et al., 2005; Wang et al., 2007; Cheng et al., 2018) and automated glycan assembly (AGA) (Panza et al., 2018; Pardo-Vargas et al., 2018; Guberman and Seeberger, 2019; Joseph et al., 2020), a wide variety of complex oligosaccharides were synthesized, yet reports on their limitations or complete failures (Guberman and Seeberger, 2019) were also observed. Thus, worldwide endeavor for the development of new other efficient glycosylation techniques is continuing and should keep going toward ultimate achievement of a generalized pathway. In the present review discussion on the current trends of glycosylation methodology-oriented research investigations, development during the past one decade will be the focus. At the end, the relative merits of the discussed methods have also been compiled in a table.

## 2 Techniques for 1,2-*Trans* Selective Glycosylation Reactions

It is well-known that 1,2-*trans* glycosidic linkage can be stereoselectively formed using an anchimeric assistance of a neighboring participating group, conventionally an *O*-acyl moiety such as *O*-acetyl (*O*Ac), *O*-benzoyl (*O*Bz), and 2-phthalimido (NPhth) (Goodman, 1967; Fife et al., 1996; Nukada et al., 1998). However, glycosyl donor with the acyl group at *C*-2-*O* position also suffers from several drawbacks like the migration of the acyl motif from *C*-2-*O* to other position and competitive formation of the 1,2-ortho ester side product (Kunz and Harreus, 1982; Sato et al., 1988; Fraser-Reid et al., 1990; Toshima and Tatsuta, 1993; Seeberger et al., 1997; Crich et al., 1999; Yu and Ensley, 2003). In the absence of traditional NGP, use of nitrile solvent and reaction temperature also modulate 1,2-*trans* selectivity, but for a given donor–acceptor pair this effect may also be very uncertain (Wulff and Röhle, 1974; Uchiro and Mukaiyama, 1996; Demchenko et al., 1997). Other pathways explored on 1,2-*trans* glycosylation reactions are discussed later.

### 2.1 Acid–Base Catalyzed 1,2-*Trans* Selective Glycosylation Reactions

**SCHEME 2:**
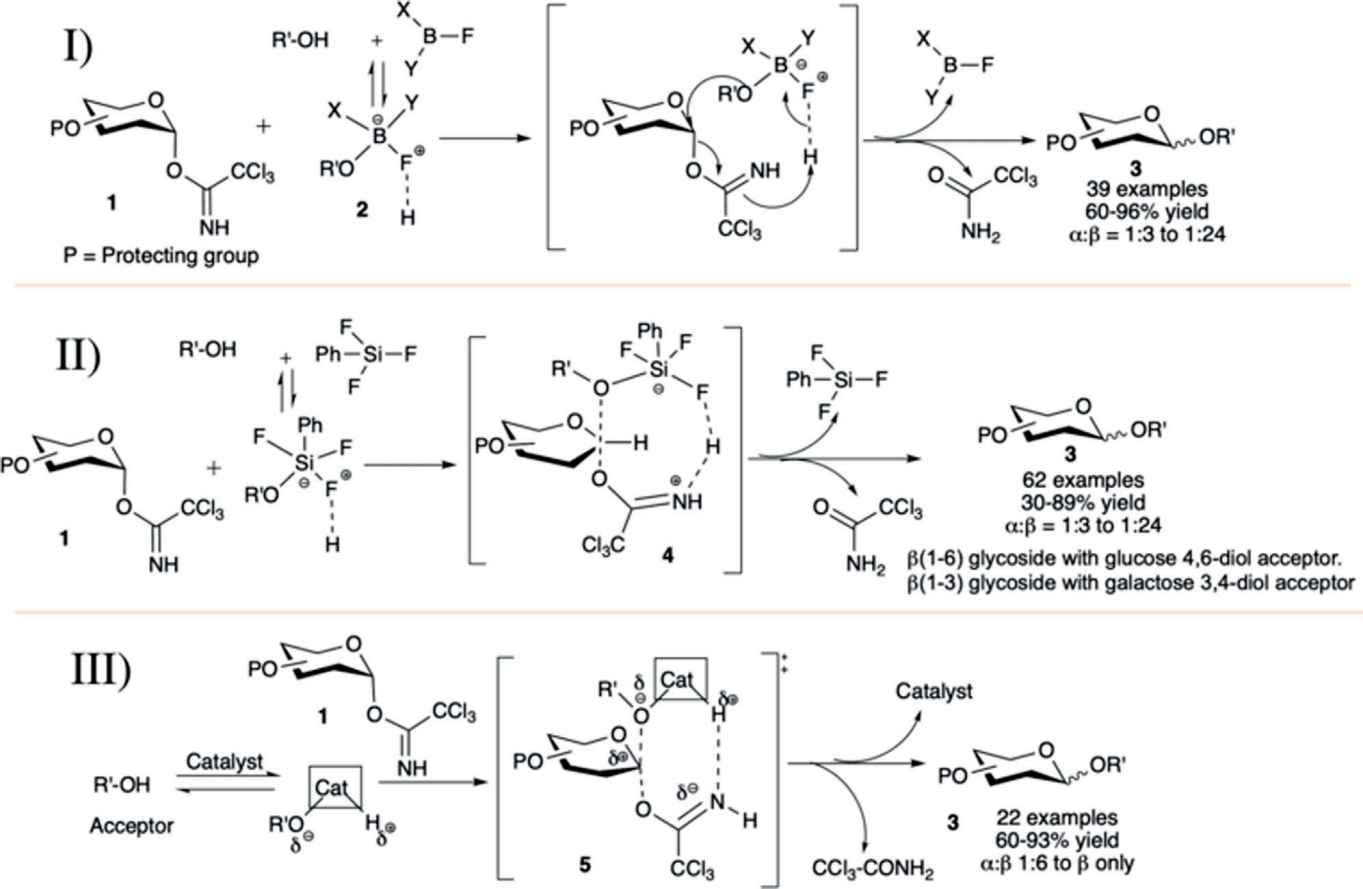
Acid–base-catalyzed 1,2-*trans* selective glycosylation reactions OTCA donors using I) phenylboron fluoride salts (PhBF_2_ or Ph_2_BF); II) phenyl silicon trifluoride salt; and III) AuCl_3_ and AuCl catalyst.

The concept of acid–base catalyzed activation of glycosyl donor and glycosyl acceptor was introduced by Kumar et al. (2011). They reported phenylboron difluoride (PhBF_2_) or diphenylboron fluoride (Ph_2_BF) catalyzed hydrogen bond (H-bond)-mediated intramolecular S_N_2-type glycosylation in general high yield and anomeric selectivity. They have shown that the reaction of trichloroacetimidate (OTCA) donor with 2-propanol using BF_3_.Et_2_O or TMSOTf was not stereoselective, but the similar reactions with less acidic boron fluoride derivatives (PhBF_2_ or Ph_2_BF) were stereoselective to produce its corresponding 1,2-*trans* glycoside. At -78°C, PhBF_2_ or Ph_2_BF did not activate and hence not decompose the glycosyl donor, but it formed a non-covalent H-bonded adduct (**2**) with glycosyl acceptor and this active adduct reacts readily with donor resulting in 1,2-*trans* glycosides. Under this condition, glycosylation reactions of different OTCA donors (**1**) without any participating neighboring groups in C-2 position with glycosyl acceptors generated mainly their corresponding 1,2-*trans* glycosides (**3**) in moderately high to excellent yields and stereoselectivity (I in Scheme 2). Reactions with secondary glycosyl acceptors were relatively poorly yielding and less stereoselective. It was evident from the results that Ph_2_BF serves as a better catalyst than that of PhBF_2_ both in terms of stereoselectivity and reaction yield, as the steric effect of the two phenyl groups supports the concerted donor activation–acceptor transfer, and the extent of stereoselectivity depends on the ease of catalyst–acceptor adduct formation. The S_N_2-type reaction mechanism between this catalyst–acceptor adduct and the 1,2-*cis* glycosyl-OTCA donor causes inversion at the anomeric center producing 1,2-*trans* glycosides.

Continuing their work on this acid–base catalyzed 1,2-*trans* selective glycosylation reactions of glycosyl–OTCA donors, Kumar et al. (2012) introduced phenyl silicon trifluoride (PhSiF_3_) as an efficient catalyst for this purpose.) The reaction of C-2 alkylated–OTCA donors (**1**) with various acceptors generated their corresponding glycosides (**3**) in excellent yield and 1,2-*trans* selectivity (II in Scheme 2) *via* the S_N_2-type mechanism. The reaction of such donor with secondary acceptor was sluggish, low yielding, and poorly stereoselective (30%, α:β = 1:3) due to the lower effective nucleophilicity of secondary sugar hydroxy groups and steric bulk of the complex with PhSiF_3_ (**4**). Coupling of galactosazide–OTCA donor with 4,6-diol acceptor resulted in regioselective glycosylation producing 1→6 linked disaccharide in good yield but poor stereoselectivity. Reaction of glucosyl/galactosyl–OTCA donor separately with 3,4-diol acceptors regioselectively produced their corresponding 1→3 glycosides in moderate to high yields and stereoselectivity (II in Scheme 2). In the case of glycosylation with fucosyl–OTCA donor and acceptor, α-disaccharide was generated exclusively *via* the S_N_1-type mechanism.

Then, Roy et al. (2015)used a combination of AuCl_3_ and phenylacetylene catalyst for 1,2-*trans* selective glycosylation of OTCA donors. Almost simultaneously the application of the catalyst AuCl_3_ itself along with AuCl was well investigated by Peng *et al.* (III in Scheme 2) (Peng and Schmidt, 2015). Under AuCl_3_-catalyzed reaction condition, reaction of per-*O-*benzylated-OCTA donor (**1**) separately with 4,6-diol and 3,4-diol acceptors, respectively, produced their corresponding 1→6 linked and 1→3 linked disaccharides in high yield and stereoselectivity. The coupling with secondary 4-OH acceptors was relatively lower yielding and poorly stereoselective. Compared to the AuCl_3_-catalyzed reactions, similar reactions with the AuCl catalyst was relatively higher yielding and stereoselective. They have also explained the stereoselective formation of 1,2-*trans* glycoside by acid–base catalysis with catalyst–glycosyl acceptor adducts as evidenced by detail NMR spectroscopic studies (Peng and Schmidt, 2015). Their study shows that the catalyst has a low affinity toward glycosyl donor but has high affinity to the hydroxy group bearing glycosyl acceptor. This affinity leads to the formation of a catalyst–acceptor adduct. Proton acidity and the oxygen nucleophilicity is increased by charge separation in this adduct. That permits the activation of the glycosyl donor and concomitant acceptor transfer in a hydrogen bond-modulated S_N_2-type transition state (**5**). More interestingly, they had observed that diol acceptors if used, can act as bidentate ligands, and then the reaction proceeds more smoothly with higher yield and even higher 1,2-*trans* selectivity. AuCl-catalyzed glycosylation reactions also exhibited similar trend, but the possible pathway for this result is different. This may be due to the expansion of linear two-coordinate gold (I) chelation to a trigonal-planar three-coordinate complex formation, or alternatively due to disproportionation of gold (I) chloride to gold (0) and gold (III) chloride.

### 2.2 Co-Operative Catalysts for Stereoselectivity Control

**SCHEME 3:**
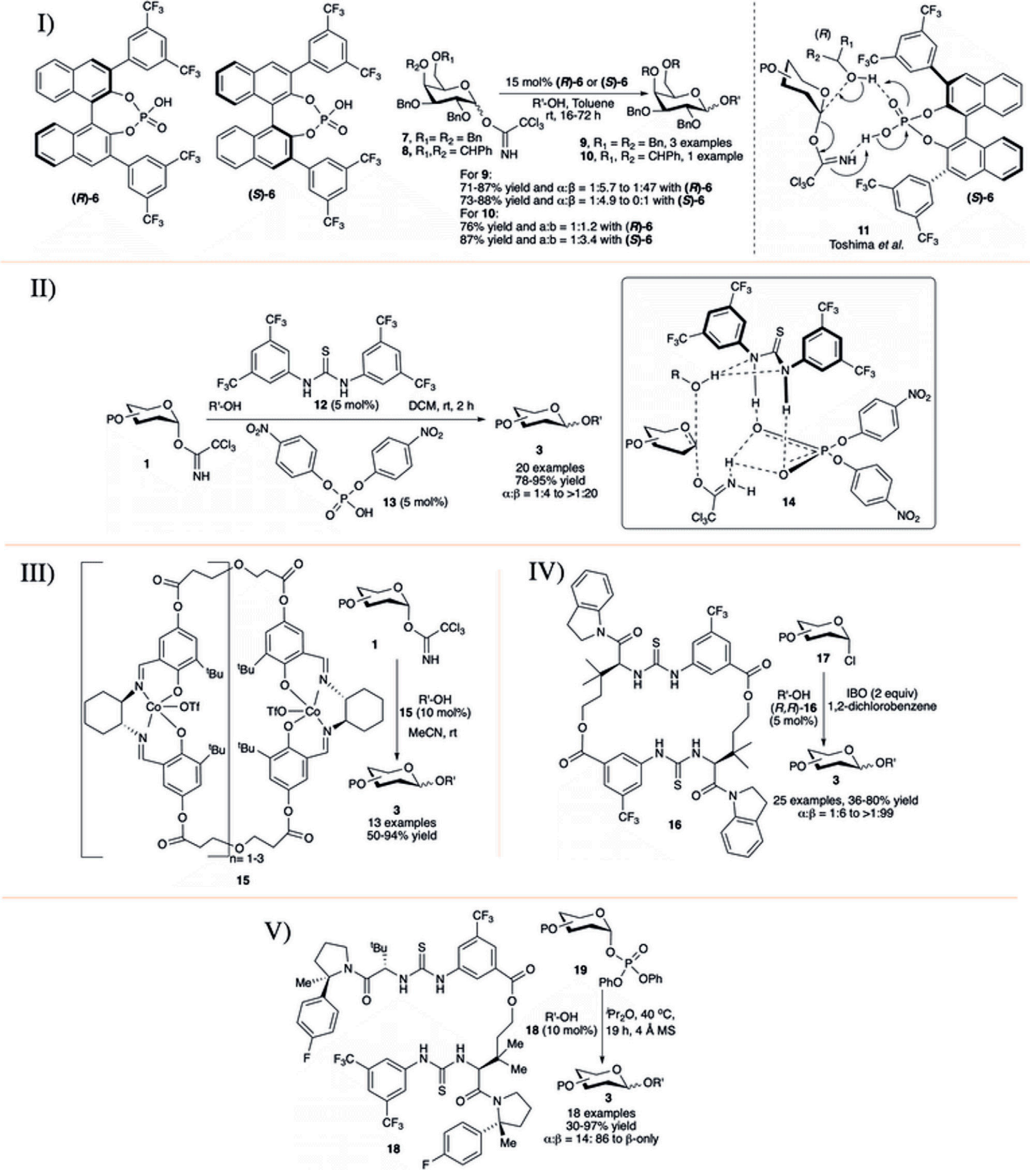
Co-operative catalysts for stereoselectivity control using I) chiral Brønsted acid (*R/S*)-**6**; II) Schreiner’s thiourea **12** and phosphoric acid **13**; III) (salen) Co-complex **15**; IV) Macrocyclic bis-thiourea **16**; and V) macrocyclic bis-thiourea catalyst **18**.

In early 2010, the Fairbanks’ group introduced a chiral BINOL-derived phosphoric acid as chiral catalysts (*R*)-**6** and (*S*)-**6** for efficient 1,2-*trans* selective activation of C-2 *O*-alkyl protected OTCA donors in toluene solvent (I in Scheme 3) (Cox et al., 2010). Initial use of achiral phosphoric acid (PhO)_2_P(O)OH as a control promoter was found to be ineffective in this transformation. Glycosylation reaction of per-*O*-benzylated galactosyl–OTCA donor **7** catalyzed by (*R*)-**6** took 48 h to reach completion producing disaccharide **9** in good yield with significantly increased 1,2-*trans* stereoselectivity (I in Scheme 3). This trend was found to be continued when (*S*)-**6** was used as the catalytic activator, and disaccharide **9β** produced almost exclusively the 1,2-*trans* anomer (I in Scheme 3). The authors did a concise study on the protecting group’s dependency on the stereoselectivity. Some protection profiles of the glycosyl donor highly alter the stereoselectivity. Simply changing the protecting groups on C-6 and C-4 from *O*-benzyl to 4,6-*O*-benzylidene **8** as the donor and using (*R*)-enantiomer of **6**, disaccharide **10** was obtained in 76% yield, but with poor β-selectivity (**10α:10β** = 1:1.2, I in Scheme 3). However, when (*S*)-**6** was used, the reaction was again found to favor the formation of the β-anomer (**10α:10β** = 1:3.4, I in Scheme 3) but not in that extent which was observed for its corresponding 4,6-di-*O*-benzyl donor **7**. Then, in 2013, the Toshima group studied the influences of different non-sugar racemic mixture of secondary chiral alcohols on the stereoselectivity of glycosylation reactions by using the same chiral catalyst **6** and found that (*R*)-**6** was more reactive than (*S*)-**6** (Kimura et al., 2013). Based on their mechanistic studies, they proposed that chiral Brønsted acid catalyst **6** stimulated both the acceptor and the OTCA donor promoting an S_N_2-type reaction with *R*-enantiomer of the racemic mixture of secondary alcohols to form a chiral transition state like **11**, which accounts for the high 1,2-*trans* selectivity.

Another approach on this ground was the use of a combination of thiourea derivative **12** and *p-*nitrophenyl phosphoric acid **13** by Schmidt *et al.* for 1,2-*trans* selective glycosylation of armed OTCA donors in non-participant solvent DCM at room temperature (II in Scheme 3) (Geng et al., 2013). The reaction of the glycosyl donor **1** and the acceptor generates activation complex **14** (inset, II in Scheme 3), facilitating an acid–base-catalyzed S_N_2-type glycoside bond formation, generating glycosides **3** in high yield and stereoselectivity. The general reaction yields and stereoselectivity for this reaction protocol were very high, especially with non-sugar acceptors.

Medina et al. (2015) published (salen) Co catalyst as a new class of bench-stable promoter **15** for the synthesis of a series of 1,2*-trans* glycosides at room temperature in MeCN (III in Scheme 3). The reactions are performed in the absence of conventional use of molecular sieves, and stereoselectivity of glycosylation using this promoter can be altered by the choice of suitable solvents like nitrile or ether solvent. Relatively low yielding but complete 1,2-*trans* selective glycosylation was observed with donors bearing a participating *O*-acyl group at the *C-*2 position, suggesting there an involvement of the neighboring group. They found α-selectivity for some of the glycosylation reactions of per*-O-*benzyl glucosyl–OTCA donor even in DCM solvent, but they did not comment on such surprising outcome.

In early 2017, Jacobsen’s group used thiourea catalyst (*R,R*)-**16** for efficient construction of 1,2-*trans* glycosides based on glycosyl chloride donor (Park et al., 2017). Reaction of per-*O*-benzylated glycosyl chlorides (**17**) with glycosyl acceptors generated their corresponding disaccharides **3** in excellent yield and stereoselectivity (IV in Scheme 3). On the contrary, the reaction with manno or rhamnopyranosyl chloride donor was 1,2-*cis* directional. Reactions with secondary acceptors were relatively low yielding with compromising stereoselectivity, especially that with per-*O-*benzylated mannopyranosyl chloride donor. The reaction protocol was equally efficient with other varieties of glycosyl chloride donors like 2-deoxy and 2-azido glycosyl chlorides for stereoselective glycoside synthesis. Theoretical studies on mechanistic pathways were consistent with a cooperative mechanism where a nucleophile and an electrophile are simultaneously activated, resulting in a highly stereospecific substitution reaction. In 2019, Jacobsen’s group reported catalytic activation of diphenyl phosphate leaving groups with bis-thiourea catalysts toward stereoselective glycosylation reactions (Levi et al., 2019). Glycosylation using glycosyl diphenyl phosphate donor **19** of primary and secondary glycosyl acceptors in the presence of 10 mol% of tailored bis-thiourea catalyst **18**, produced their corresponding disaccharides **3** in excellent yields and β-anomeric stereoselectivity (V in Scheme 3). This glycosylation technique is equally efficient with simple hydroxylic amino acids, like serine, threonine, and hydroxyproline dipeptides, and tripeptide as acceptors and even with thiol nucleophile cysteine, although the inadequate solubility of the tripeptide under the reaction conditions resulted in lower reactivity. Galactosylation of compounds like clindamycin (relatively low yielding, 34%, α:β = 18:82) and quetiapine, bearing basic nitrogen functionality was also achieved, suggesting that elaboration toward medicinally active small molecules’ synthesis is also possible. Great scope was observed with protic nucleophiles, but carbon-centered nucleophiles like allyltrimethylsilane was found unreactive under the catalytic conditions.

### 2.3 Metal-Catalyzed 1,2-*Trans* Selective Glycosylation Reaction

**SCHEME 4:**
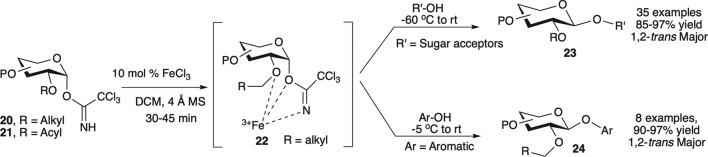
Iron (III) chloride modulated 1,2-*trans* selective glycosylation reactions of trichloroacetimidate donors.

Mukherjee et al. (2016) utilized the first-row transition metal Lewis acid iron (III) chloride (0.1 equivalent) as an inexpensive and environmentally benign catalyst for 1,2-*trans* glycosylation of C-2-*O*-alkyl protected glycosyl–OTCA donor. Reaction of C-2-*O*-alkyl protected (**20**) or C-2-*O*-acyl protected (**21**) OTCA donors with a variety of acceptors at −60°C to room temperature generated their corresponding 1,2-*trans* glycosides (**23**) in high to excellent yields and stereoselectivity (Scheme 4). For C-2-*O*-alkyl protected glycosyl OTCA donors (**20)**, the reaction is believed to proceed through a 7-membered chelate complex (**22**) where a bulky pendant C2–O–[Fe]NCOCCl_3_ blocks the α-face and drives the incoming nucleophile to attack from β-side resulting in 1,2-*trans* glycoside (**23**), whereas C-2-*O*-acyl protected glycosyl donors (**21**) proceed through the traditional NGP pathway. Reaction of per-*O*-alkyl protected donors with electron rich phenols at relatively higher temperature (−5°C to room temperature) produced their corresponding β-*O-*aryl glycosides (**24**) in excellent yields. They used this reaction protocol for efficient semi orthogonal activation of OTCA donor over thioglycosides, and later a combination of NIS/FeCl_3_ was used to activate thioglycoside donor as well. A model trisaccharide was also synthesized by FeCl_3_ catalyzed one-pot activation of OTCA donor over thioglycoside donor followed by thioglycoside activation using NIS/FeCl_3,_ and this protocol was further used in other higher oligosaccharide syntheses (Mukherjee and Ghosh, 2017).

### 2.4 Other Methods of 1,2-*Trans* Selective Glycosylation Reactions

**SCHEME 5:**
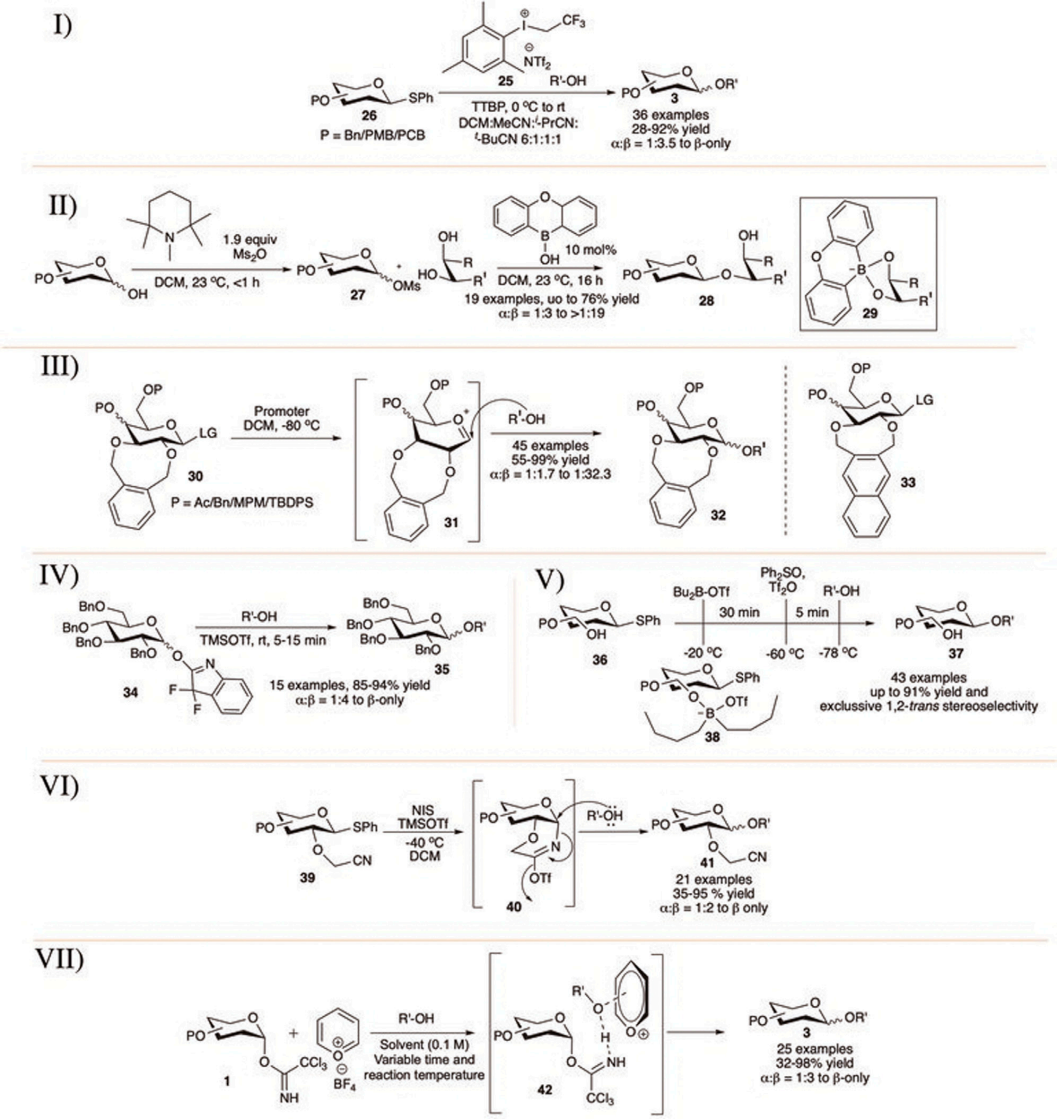
1,2-*trans* selective glycosylation reaction using I) aryl(trifluoroethyl)iodoniumtriflimide catalyst; II) borinic acid; III) 2,3-*O*-xylene protection; IV) *O*Fox donors; V) unprotected sugar derivatives; VI) 2-cyanomethyl ether-protection; and VII) pyrylium salt catalyst.

Chu et al. (2015) showed that armed thioglycosides (**26**) can be activated using aryl(trifluoroethyl)iodonium triflimide (**25**) in 2:1 DCM/pivalonitrile or in a solvent mixture of DCM, acetonitrile, isobutyronitrile, and pivalonitrile (6:1:1:1) at 0°C toward glycosylation reactions in good yield and moderate to excellent selectivity (I in Scheme 5). Glycosylation reactions of primary acceptors separately with globally PMB-protected glucoside donor provided disaccharide products with higher levels of β-selectivity than the corresponding per-*O*-benzylated analog but proceeded in much lower yield. They have shown that glycosylation reaction using three commonly used thiophilic promoters (NIS/TfOH; BSP/Tf_2_O/TTBP; and NBS/TfOH) under the present optimal mixed solvent composition and similar condition results their corresponding disaccharide with loss of stereoselectivity. These results indicate that apart from the simple solvent participation during glycosylation, this aryl(trifluoroethyl)iodonium triflimide in combination with these nitrile solvents is indeed activating the thioglycosides through a different, more selective, pathway than other commonly used promoters.

D'Angelo and Taylor (2016) reported a detailed study of oxaboraanthracene-derived borinic acid-mediated and base-promoted glycosylation of armed glycosylmesylate donors (**27**) for 1,2-*trans* selective glycosylation. They have shown that these glycosylmesylate donors can be prepared *in situ* from the reaction between the corresponding glycosyl hemiacetals and methane sulfonic anhydride at room temperature within 1 hour under conditions of base catalysis, and these also serve well as glycosyl donor in organoboron-catalyzed glycosylation (II in Scheme 5); 1,2- or 1,3-*cis* diols were used as glycosyl acceptors, and the reaction proceeds predominantly with a regioselective preference for the equatorial hydroxy group on the saccharide acceptor. Interestingly, from a reaction of the glucofuranosyl donor the product was generated with a preference for activation of the secondary 5-OH group over the primary 6-OH group (8:1 regioselectivity), possibly *via* the 3,5-*O*-borinate. A modest to high α-selectivity was observed when the reactions were carried out in the absence of the organoboron catalyst and that clearly demonstrated the stereochemical influence employed by the organocatalyst. Extensive kinetic and mechanistic studies provide evidence in support of an associative mechanism where an intermediate boronate ester (**29**) is formed through the reaction of the diarylborinic acid and the glycosyl acceptor. This boronate ester undergoes glycosylation with the more reactive α-anomer of the glycosylmesylate predominantly forming the 1,2-*trans* linked products (**28**).

Then, Yagami et al. (2017) introduced eight-(2,3-*O*-xylylene) membered fused rings to protect cyclically the C-2 and C-3 hydroxy groups of glycosyl donor toward 1,2-*trans* selective glycosylation reaction (III in Scheme 5). Glycosylation reaction of various 2,3-*O-*xylene protected donor (**30**) separately with different glycosyl acceptors generated mainly their corresponding β-glycosides (**32**) in high to excellent yields and stereoselectivity. β-selectivity of the glycosylation reaction was affected by the nature of substituents present on the C-4 and C-6 positions. An electron withdrawing group at either or both two positions decreased the stereoselectivity, whereas the electron donating protecting group increased the β-selectivity. Low temperature NMR spectra and theoretical studies suggested that the reaction proceeds through an oxocarbenium intermediate (**31**), where an eclipsing interaction between the C-2-pseudo-equatorial xyloxy group and the incoming nucleophile disfavors 1,2-*cis* attack. Later on, in 2020, they modified this with 2,3-*O-*napthalenedimethyl group protected glycosyl donor (**33**) for similar purpose and extended this work for efficient synthesis of diglycosyl diacylglycerols (DGDGs) (Yagami et al., 2020).

In the same year, Demchenko’s group introduced 3,3-difluoro-3*H*-indolyl (*O*Fox) imidate as a good leaving group for glycosylation reaction of both armed and disarmed donors with 1,2-*trans* selectivity (Nigudkar et al., 2017). These *O*Fox-imidate donors can be derived easily from their corresponding glycosyl bromide or thioglycoside or even from glycosyl hemi-acetals in excellent yields. Glycosylation reactions of *O*Fox donor (**34**) with different acceptors using 0.05 equivalent of TMSOTf at −78°C gave glycoside **35** in excellent yields and 1,2-*trans* selectivity (IV in Scheme 5). Reactions with secondary acceptors were moderately 1,2-*trans* selective. This *O*Fox can also be activated easily within 5 min using 0.1 equivalent of BF_3_.Et_2_O or 0.2 equivalent of Cu(OTf)_2_.

Liu’s group reported dialkylboryl triflate as an *in situ* masking reagent of the free hydroxy group toward stereoselective glycosylation with thioglycoside donor carrying one-to-three unprotected hydroxy groups (Le Mai Hoang et al., 2017). In this minimalist approach, first the hydroxy group of thioglycoside donor (**36**) is temporarily masked with dibutyl borane (**38**) and then thioglycoside is pre- activated with diphenyl sulfoxide (Ph_2_SO) and Tf_2_O followed by the reaction with glycosyl acceptor ultimately resulting the corresponding glycosides (**37**) in excellent yields and stereoselectivity (V in Scheme 5). General trend of high 1,2-*trans* selectivity was observed when there was either C2-OH or C3-OH available but was strongly reduced when there was an acetyl protection adjacent to the free hydroxy group (α:β = 1:4.5 or 1:6), C6-OH was present (α:β = 1.2.5) or the glucosyl donor carries three free hydroxy groups (α:β = 1:1.7).

In 2020, Molla et al. (2020) reported efficient synthesis of 1,2-*trans* glycosides (**41**) using cyanomethyl ether protection at the C2 position of thioglycoside donors (VI in Scheme 5). The reactions of various 2-cyanomethyl ether protected thioglycosides (**39**) separately with various acceptors using NIS/TMSOTf at −40°C generated their corresponding glycosides (**41**) in moderate to very high yields and stereoselectivity. Particularly, reactions with sterically hindered secondary sugar acceptor were low yielding. Detailed theoretical and control experimental studies suggest that the cyanomethyl ether protection acts as a participating group from 2-position and form a six-membered imine-type intermediate (**40**) directing the incoming nucleophile from β direction.

Very recently Nielsen et al. (2022) reported the pyrrylium catalyst (H_3_Pypy) catalyzed β-selective glycosylation of trichloroacetimidate donors. Reaction of reactive carbohydrate or phenol acceptors with α-trichloroacetimidate donor (**1**) in the presence of 0.1 equivalent of H_3_Pypy catalyst generated their corresponding β-glycosides (**3**) in good yield and stereoselectivity (VII in Scheme 5). The reaction with secondary acceptor was low yielding and not stereoselective (40%, α:β = 1:1). Similar reactions with β-trichloroacetimidate donor using Me_3_Pypy catalyst in Et_2_O solvent was in some extent α-directional. The control experiment suggested that the reactions are most likely proceed through an S_N_2-like substitution on the activated complex (**42**)**.**

### 2.5 β-Linked Glycosylation Based on 2-Deoxysugars

2-Deoxyglycosides are structural elements of microbial secondary metabolites having important biological effects, including antitumor, antibiotic, and cardiac activities. Construction of β-linked 2-deoxyglycosides is one of the most difficult glycosidic bonds to synthesize (Hou and Lowary, 2009; Crich, 2011; Borovika and Nagorny, 2012). The lack of a stereo directing substituent adjacent to the anomeric center renders many of the conventional approaches for the construction of β-glycosidic bonds relatively unselective.

**SCHEME 6:**
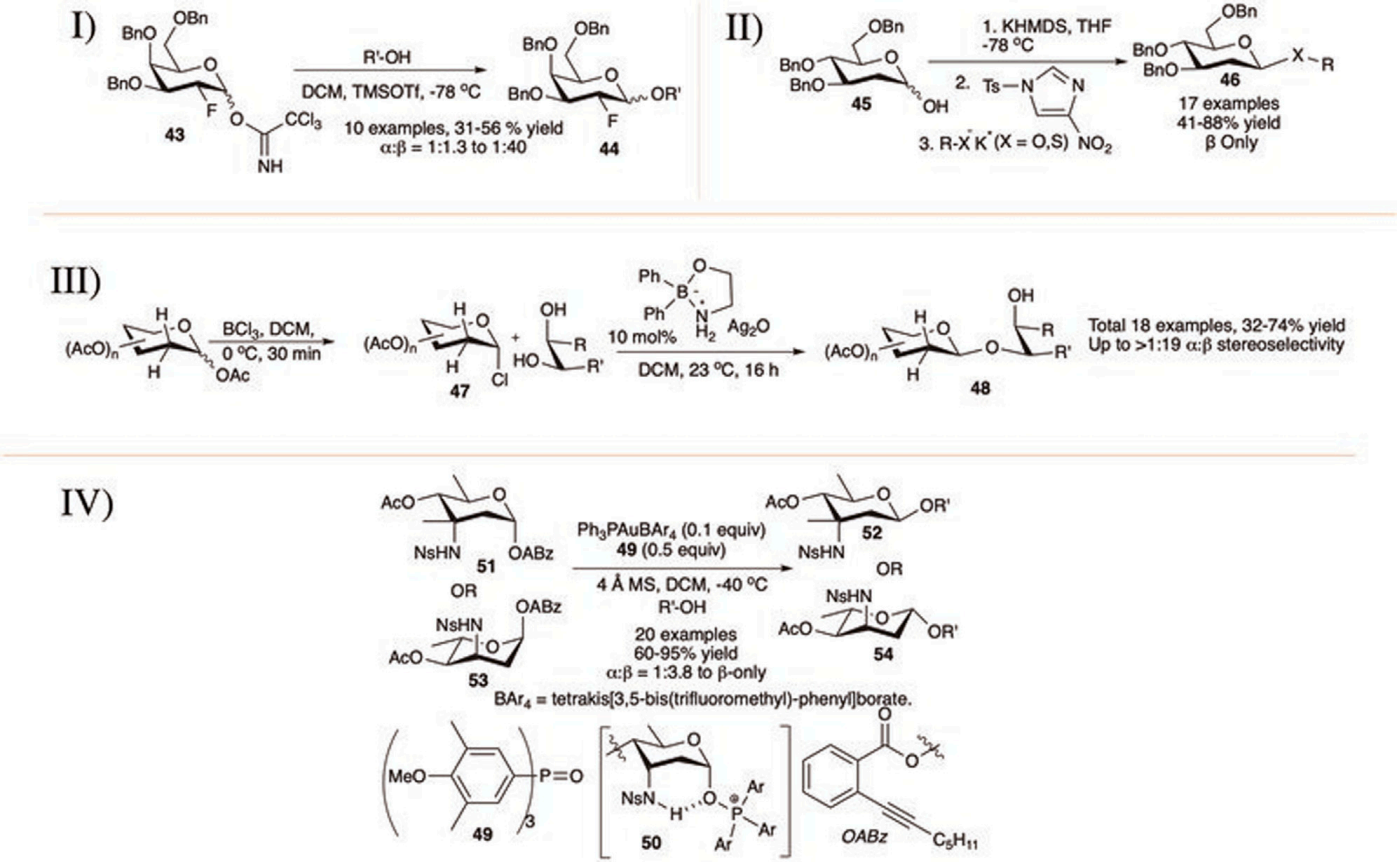
β-linked glycosylation reaction of 2-deoxy glycoside donors using I) 2-fluoroglycosyl donors; II) KHMDS and *N*-tosyl 4-nitroimidazole promoter; III) organoboron-catalyst; and IV) phosphine oxide **49** catalyst.

Durantie et al. (2012) reported β-linked glycosylation with 2-deoxy-2-fluoro trichloroacetimidate donor (I in Scheme 6). Thus, tri-*O-*benzyl-2-deoxy-2-fluoro-d-galactopyranosyl OTCA donor (**43**) coupled separately with different primary pyranoside acceptors generating their corresponding disaccharides (**44**) in moderate yield but with excellent 1,2-*trans* selectivity. Reactions with non-sugar alcohol acceptors were higher yielding and more stereoselective, whereas reactions with secondary sugar acceptors were low yielding and poorly stereoselective.

The Bennet’s group activated 2-deoxysugar hemiacetals for glycosylation with sulfur or oxygen nucleophile in the presence of KHMDS and *N*-tosyl 4-nitroimidazole *via in situ* generations of glycosyl sulfonates (Issa et al., 2013). Coupling between 2-deoxy hemiacetal (**45**) and potassium thio-salt of the phenol or sugar acceptor produced their corresponding β-2-deoxythioglycoside or β-2-deoxydisaccharide (**46**), respectively (II in Scheme 6). The same process has also been used to synthesize aryl 2-deoxyglycosides in good yield and exclusive β-selectivity. The presence of diglyme as a solvent increased the reaction yield without hampering stereoselectivity as such solvent is capable of better coordination of the counter ion. They changed the sulfonylating reagent to a more potent *p*-toluenesulfonic anhydride for successful reaction with secondary sugar acceptors, but this did not work for acetonide-protected acceptors.

With their work on organoboronic acid derivative catalyzed regioselective and stereoselective glycosylation Taylor’s group in 2014 reported β-linked glycosylation using 2-deoxy and 2,6- dideoxy glycosyl chloride donors (**47**) of *cis*-1,2- and 1,3-diol acceptors (III in Scheme 6) (Beale et al., 2014). Glycosyl chlorides were generated from their corresponding glycosyl acetates by simple treatment with BCl_3_ in DCM. Thus, generated glycosyl chloride donors were then activated using Ag_2_O in the presence of organoborinic acid ester toward regio- and stereoselective glycosylation generating 2-deoxyglycosides (**48**) in good to high yields and stereoselectivity. Glucopyranosyl 4,6-diol acceptor regioselectively reacted through 6-OH group producing the corresponding 1→6 glycosides, and 2,3,4-triol acceptors regioselectively produced the corresponding 1→3 glycosides.

In 2019, Zeng et al. (2019) *.* reported a novel strategy for the construction of the less investigated β-glycosidic bonds of rare amino-sugars, which constitute the core structure of several biologically important antibiotics. The axial sulfonamide group present in C-3 position of 3,5-*trans*-3-amino-2,3,6-trideoxy sugars (3,5-*trans*-3-ADSs) acts as a hydrogen-bond (H-bond) donor and repurposes sub-stoichiometric phosphine oxide (**49**) as an exogenous nucleophilic reagent (exNu) to establish an intramolecular H-bond between the former and the derived α-oxyphosphonium ion. This pivotal interaction stabilizes the α-face-covered intermediate (**50**) and inhibits the formation of the more reactive β-intermediate, yielding eventually the β-glycosides. A wide range of substrates was exemplified under this H-bond-assisted exNu effect-derived glycosylation protocol producing good to excellent β-selective 2,6-dideoxyglycosides. The reaction of 3,5-*trans* 2,6-dideoxy alkyl benzoate donor (**51**) or (**53**) separately with various acceptors furnished their corresponding disaccharides (**52**) or (**54**) in good to excellent yield and stereoselectivity (IV in Scheme 6). The efficacy of the present strategy was further demonstrated by the structural modifications of natural products and drugs containing 3, 5-*trans*-3-ADSs, as well as the synthesis of a trisaccharide unit present in avidinorubicin. The less sterically hindered and electron-rich primary alcohols showed lower selectivity.

## 3 Techniques for 1,2-*Cis* Glycosylation Reactions

Unlike the 1,2-*trans* glycosylation techniques, the formation of 1,2-*cis*-glycosidic linkages is typically much more challenging and requires the absence of neighboring group participation at the C-2 position. Naturally, the most popular choices for C-2 OH protection are ether linkages in the case of 1,2-*cis* glycosylation reactions. This type of glycosylation cannot ensure stereoselectivity and usually leads to a mixture of anomers as products, although the α-product is favored by the anomeric effect. Often, calibration of reaction solvent proves to be a useful tool maintaining α:β ratio of products in favor of the 1,2-*cis* one. But tedious isomeric separation processes are unavoidable on numerous occasions. A number of research works have been published so far to overcome this long-standing hurdle of 1,2-*cis* glycosylation and every detail of those have been very efficiently reviewed by Nigudkar and Demchenko (2015), and here mainly the reports those have been published thereafter will be discussed in details.

### 3.1 Urea/Thiourea Catalyzed 1,2-*Cis* Selective Glycosylation Reactions

**SCHEME 7:**
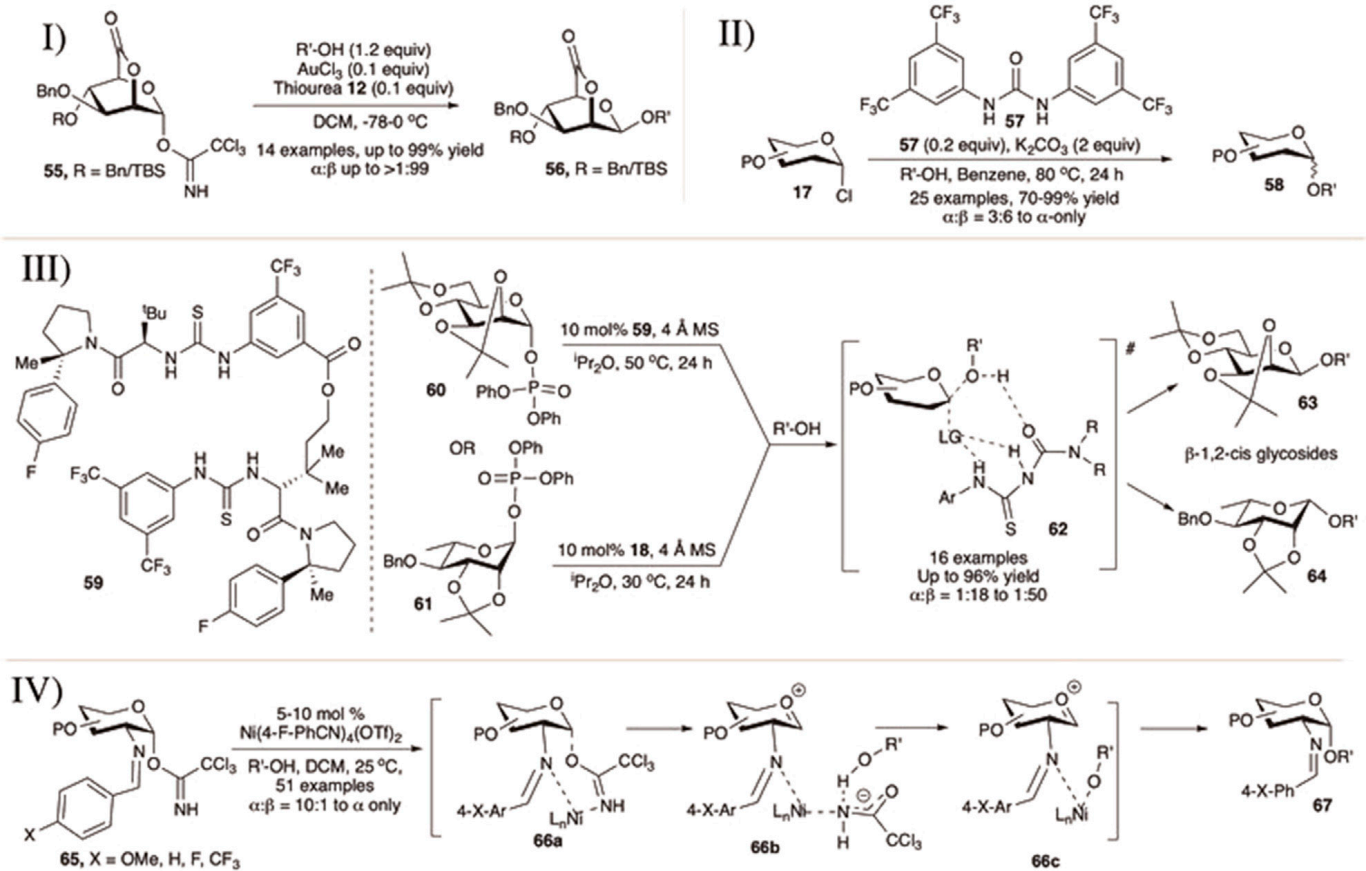
I) 1,2-*cis* selective mannosylation using 2,6-lactone donor; II) urea-catalyzed α-selective glycosylation reactions of glycosyl chloride donors; III) glycosyl phosphate for 1,2-*cis* selective glycosylation of D-mannose and L-rhamnose using macrocyclic bis-thiourea derivatives; and IV) nickel (II)-catalyzed formation of 1,2-*cis*-2-aminoglycosides.

Hashimoto et al. (2016) published the 1,2-*cis* mannosylation technique utilizing 2,6-lactone derivatives of mannopyranosyl OTCA donor using AuCl_3_ and thiourea (**12**) as the combination catalyst (I in Scheme 7).) They have also studied the effect of glycosyl donor concentration on the stereochemical outcome of glycosylation reactions. The reaction of 2,6-lactone–OTCA donor (**55**) with various glycosyl acceptors generated their corresponding disaccharides (**56**) in good yield and stereoselectivity (I in Scheme 7). The results of reaction between 2,6-lactone donors and secondary sugar acceptor vary depending on the protection group profile and concentration. For all the reactions, they observed a clear tendency of 1,2-*cis* glycoside formation in concentrated solution *via* S_N_2-like reaction pathway whereas α-glycosides were formed in diluted solution through the S_N_1-like mechanism. The reactions proceeded mainly with concentration-dependent stereo inversion, with the glycosyl cation generated from the donor being sterically β-directing.

Sun et al. (2016) reported urea (**57**)-catalyzed H-bond activation of glycosyl chlorides (**17**) toward stereoselective glycosylation reactions in excellent yields and α-stereoselectivity (II in Scheme 7). Reaction of different per-*O-*benzylated d-galactopyaranosyl/d-mannopyranosyl/L-rhamnopyranosyl chloride donors separately with various acceptors using 0.2 equivalents of urea derivative (**57**) and 2.0 equivalents K_2_CO_3_ in refluxing benzene generated their corresponding α-glycosides (**58**) in excellent yields and stereoselectivity. Similar reaction with per-*O-*benzyl glucopyranosyl chloride donors were high yielding but less stereoselective (71–91% yield, α:β = 2:1–3:1). This problem of low α-stereoselectivity of glycosylation with per-*O*-benzylated glucosyl donors was greatly improved by the addition of 1.5 equivalents of tri-(2,4,6-trimethoxyphenyl)phosphine (TTMPP). The presence of benzoate or NPhth functionality at C-2 position drives the reaction course in the usual direction *via* NGP.

Recently, in 2020, Jacobsen’s group used the catalyst (**18**) and (**59**) for protecting group-dependent 1,2-*cis* selective glycosylation using mannose and rhamnose diphenyl phosphate donors of amino acids, carbohydrates, and complex natural products (Li et al., 2020). Coupling of 2,3:4,6-bisacetonide-protected mannopyranosyl diphenylphophate donor (**60**) with different acceptors using 10 mol% of catalyst (**59**) produced the corresponding disaccharides (**63**) in excellent yield and 1,2-*cis* stereoselectivity (III in Scheme 7). The reactions of the L-rhamnopyranosyl donor (**61**) were even more efficient compared to the similar reaction with d-mannopyranosyl donor. When (**18**), other enantiomer of the catalyst (**59**) was used, the resulting disaccharides (**64**) were obtained in greater yields and stereoselectivity (III in Scheme 7). Simple alkyl-protections such as per-*O-*benzyl or per-*O*-methyl resulted in mainly unselective coupling reactions for mannopyranosyl donors. In contrast, in almost all cases, TMSOTf promoted coupling yielded α-anomers as the major glycosylated products, consistent with the expected inherent preference for α-product in mannosylations, thus confirming the participation of catalyst toward stereochemical outcome rather than strain in protecting groups. It was also evident from NMR and controlled experiments that the catalyst plays an important role in the formation of the complex transition state (**62**) directing the glycosylation toward 1,2-*cis* fashion.

### 3.2 Transition Metal-Catalyzed 1,2-*Cis* Selective Glycosylation Reactions

Selective construction of 1,2-*cis* amino glycosides remains problematic because of the necessity of the non-participating group at C-2 of the donor. Nguyen’s group has made significant advances to overcome these limitations using cationic nickel (II) catalysts (IV in Scheme 7) (Mensah and Nguyen, 2009; Mensah et al., 2010). Mensah and Nguyen (2009) reported activation of C (2)-*p*-methoxybenzylidene protected 2-amino glycosyl-OTCA donor (**65**) in the presence of primary/secondary or tertiary acceptors using 5–10 mol% of Ni(4-F-PhCN)_4_(OTf)_2_ at room temperature generating their corresponding 2-amino-2-deoxyglycosides (**67**) in general high yields and 1,2-*cis* stereoselectivity. Later, they examined the effect of various substituents at 4-position of the phenyl ring of the C (2)-benzylidene protected 2-amino glycosyl–OTCA donors for the similar glycosylation reactions (Mensah et al., 2010). Changing the *p*-OMe group with electron deficient “*p*-F” or “*p*-CF_3_” does not have any general significant effect on the outcome of the reaction both in terms of reaction yields and stereoselectivity, except for the reaction with α-methyl glucopyranosyl secondary acceptor. For such reactions, changing *p*-OMe with “*p*-F” increases reaction yield and stereoselectivity and this trend was continued even better with “*p*-CF_3_.” They have also applied this technique for successful synthesis of multiple trisaccharides, tetrasaccharides, glucosamine-α-(1→4)-linked glucuronic acid derivatives of heparin, pseudo-glycoside core of GPI anchors, and 2-amino glycosyl-serine or threonine derivatives as well. Their proposed mechanistic pathways suggested that the presence of C (2)-benzylidene protection functionality is essential for α-selective stereochemical outcome. Thus, the reaction probably proceeds *via* interaction between the C (2)-*N*-nickel metal-hydroxy group of the incoming nucleophile and anomeric center resulting in chelate complexes **66a–66c** where the α-orientation of the C (1)-trichloroacetimidate group on glycosyl donor is necessary.

### 3.3 Chiral Auxiliary Derived 1,2-*Cis* Stereoselective Glycosylation Reactions

**SCHEME 8:**
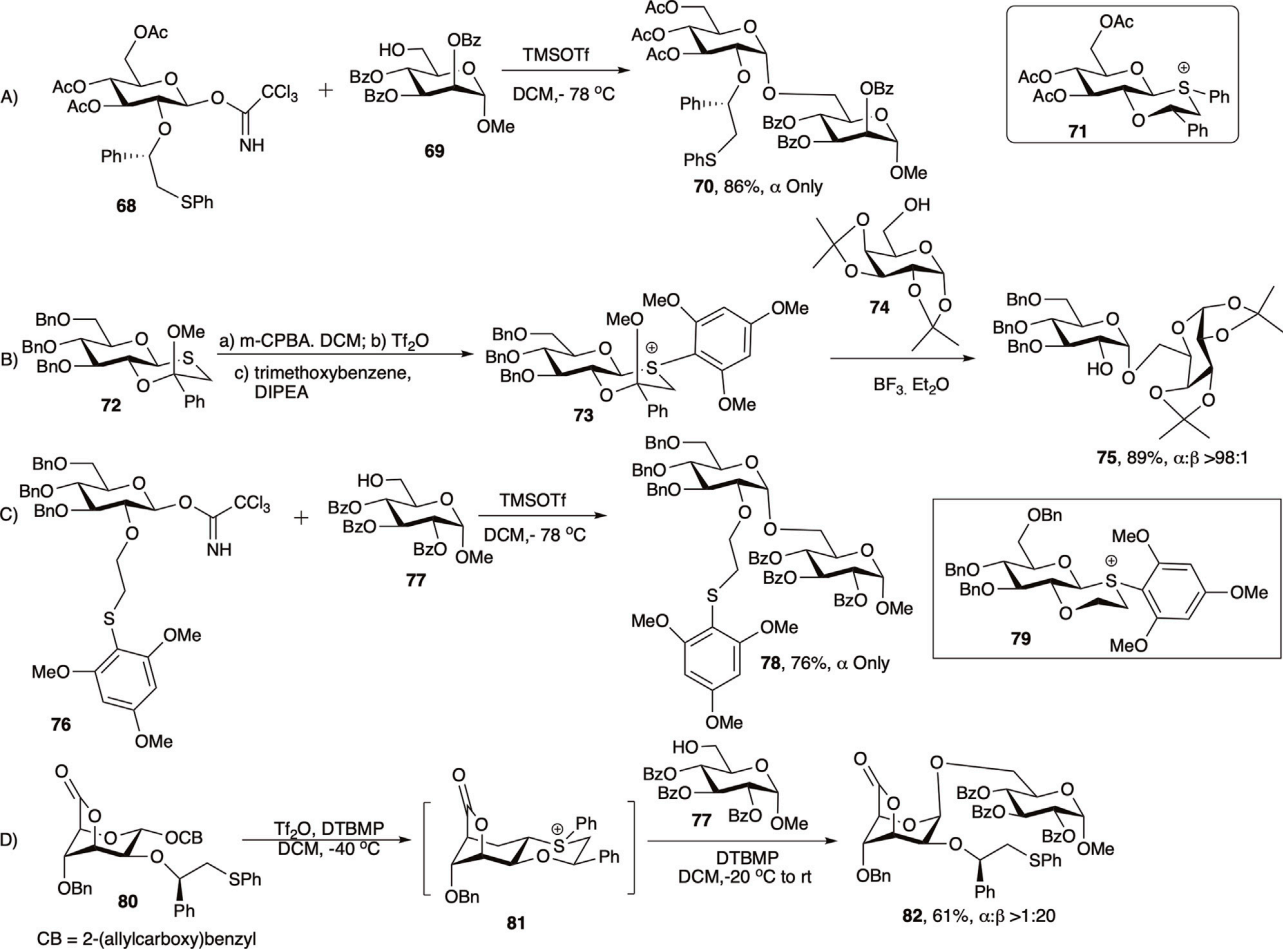
1,2-*cis* glycosylation reactions using the secondary auxiliary group.

Mensink and Boltje (2017) and Jeanneret et al. (2020) in 2020 reviewed the application of C-2 chiral auxiliary on 1,2-*cis* glycosylation in much details, and here only a brief outlook on the evolution of this C-2 chiral auxiliary groups will be discussed. In 2005 Boons and co-workers developed a chiral auxiliary-based glycosylation technique with a participating group at C-2 of the glycosyl donor that participated from the opposite face of the ring resulting in 1,2-*cis* linked glycosides (Kim et al., 2005a). The second generation chiral auxiliary, (*S*)-phenylthiomethylbenzyl ether moiety was developed to further enhance the 1,2-*cis* stereoselectivity of the reaction (Kim et al., 2005b; Fascione et al., 2009; Boltje et al., 2011). Coupling of glycosyl donor (**68**) with acceptor (**69**) afforded exclusively 1,2-*cis* linked disaccharide (**70**) in 86% yield (entry a, Scheme 8). It was assumed that this type of moiety would form a chair-like, and hence a more stable *trans*-decalin-like intermediate (**71**), and the stereo-outcome would be more selective and efficient. In this case, the (*S*)-phenyl group will avoid the axial position to circumvent unfavorable 1,3-diaxial interactions and will occupy the equatorial position.

More recently, as a simplification of this approach, Boons and co-workers adopted a different approach toward synthesis of 1,2-*cis* glycosides (Fang et al., 2012). Thus, the coupling of thioglycoside donor (**72**) with acceptor (**74**) proceeds through the intermediate (**73**), resulting in their corresponding disaccharide (**75**) in 89% yield and almost exclusive 1,2-*cis* stereoselectivity (entry b, Scheme 8). A few years later Fairbanks *et al.* on the other hand reported a similar glycosylation based on a fully armed donor (**76**) bearing a 2-*O*-(trimethoxybenzenethiol) ethyl ether protecting group, which in reaction with acceptor (**77**) generated the corresponding disaccharide (**78**) in 76% yield and complete 1,2-*cis* stereoselectivity through intermediate (**79**) (entry c, Scheme 8) (Singh et al., 2015). In 2016, Boltje’s group used chiral auxiliary-derived NGP toward efficient construction of 1,2-*cis* mannopyranosides by the use of ^1^C_4_ conformation (Elferink et al., 2016). Reaction of allylcarboxybenzyl donor (**80**) bearing (*R*)-phenylthiomethylbenzyl ether chiral auxiliary at 2-position reacted with acceptor (**77**) proceeding *via trans*-decalin type intermediate **81** and generated the corresponding disaccharide **82** in moderate yield and excellent 1,2-cis stereoselectivity (entry d, Scheme 8).

### 3.4 Boronic Ester Modulated 1,2-*Cis* Stereoselective Glycosylation Reactions

**SCHEME 9:**
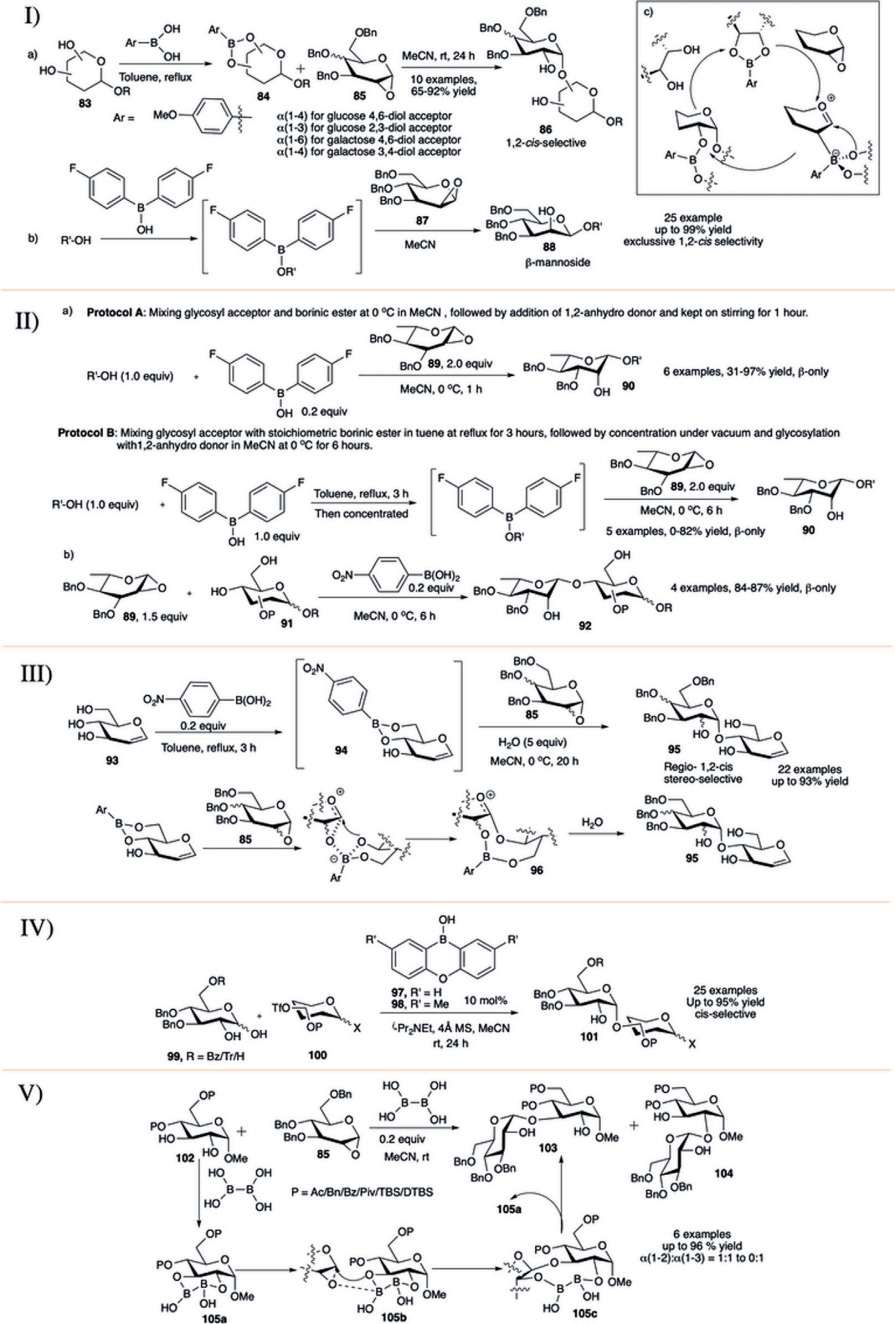
I) 1,2-*cis* selective glycosylation reaction using glycosyl acceptor derived boronic ester catalysts; II) organoboron-catalyzed β-L-rhamnosylation; III) boronic-acid-catalyzed regioselective 1,2-cis glycosylation reactions of unprotected sugar acceptor; IV) synthesis of 1,2-cis-glycosides by anomeric O-alkylation with organoboron catalysis; and V) diboron-catalyzed regio and 1,2-cis-α-stereoselective glycosylation reaction.

In 2015, a relatively new concept toward 1,2-*cis* glycosylation was introduced by Takahashi and Toshima utilizing glycosyl acceptor-derived boronic ester catalyst (Nakagawa et al., 2015). They investigated the formation of 1,2-*cis* glycosidic bond using 1,2-anhydro sugar through reversibly binding boronic esters in *cis-*1,2- or 1,3-diol of the glycosyl acceptor (**83**). Glycosylation of boronic ester (**84**) with per-*O-*benzylated 1,2-anhydroglycoside donor (**85**) resulted in the corresponding disaccharide (**86**) in high yield and exclusive 1,2-*cis* stereoselectivity (entry a, I in Scheme 9). They used this reaction procedure toward successful construction of various 1,2-*cis* linked glycosides with glucose and mannose derived boronic esters. The reaction of 1,2-anhydro sugar (**85**) with galactose-4,6-diol acceptor regioselectively generated their corresponding α(1→6)-disaccharide, whereas the glucose-4,6-diol acceptor generated α(1→4)-glycosides. Similarly, reaction between (**85**) and glucose 2,3-diol acceptor regioselectively produced their corresponding α(1→3)- glycosides, and galactose 3,4-diol acceptor produced α(1→4)-glycosides. The mechanistic pathway forming 5-membered boron complex has been shown in entry c of I in Scheme 9. They have extended this concept of glycosyl acceptor-derived borinic ester for efficient construction of β-mannosides (**88**) from 1,2-anhydromannose donor **87** and mono-ol acceptors (entry b, I in Scheme 9) (Tanaka et al., 2016a). The reaction was very high yielding (up to 99%), completely 1,2-*cis* stereoselective and successfully used for total synthesis of acremomannolipin A. In a different publication they also used this protocol for efficient total synthesis of GSL-1 and GSL-1′ (Tanaka et al., 2016b).

Nishi et al. (2018) used glycosyl acceptor-derived boronic or borinic ester for stereospecific rhamnopyranosylation of 1,2-anhydro-L-rhamnopyranose donor and mono-ol acceptors. These reactions proceeded smoothly following either one of the two protocols to provide the corresponding β-L-rhamnopyranosides with complete stereoselectivity in moderate to high yields. Coupling of 1,2-anhydro-L-rhamnopyranose donor (**89**) and different acceptors following protocol A (mixing glycosyl acceptor and borinic ester at 0°C in MeCN , followed by the addition of 1,2-anhydro donor and kept on stirring for 1 h) exclusively produced their corresponding β-disaccharides (**90**) in moderate to excellent yields (entry a, II in Scheme 9). Unfortunately, when the reaction was performed with sterically hindered secondary acceptor, in some cases the outcome was complete recovery of the glycosyl acceptor. In some other cases changing to reaction protocol B (mixing glycosyl acceptor with stoichiometric borinic ester in toluene at reflux for 3 h, followed by concentration under vacuum and glycosylation with 1,2-anhydro donor in MeCN at 0°C for 6 h) increases the reaction yield. Glycosylation reaction with various 4,6-diol acceptors (**91**) in the presence of 4-nitrophenylboronic acid generated their corresponding 1→4 disaccharides (**92**) in excellent yields and complete 1,2-*cis* stereoselectivity (entry b, II in Scheme 9). Mechanistic studies of the borinic ester mediated glycosylation along with DFT calculations were consistent with a concerted S_N_i mechanism with an exploded transition state. They used this glycosylation method for successful synthesis of a trisaccharide associated with *Streptococcus pneumoniae* serotypes 7B, 7C, and 7D.

In 2018, Tanaka et al. (2018) developed water mediated *p*-nitrophenylboronic acid catalyzed highly regio- and 1,2-*cis* stereoselective S_N_i-type glycosylation with 1,2-anhydro donors and unprotected sugar acceptors (**93**, III in Scheme 9). The reaction advances through the boron-mediated carbohydrate recognition along with a S_N_i-type mechanism resulting in highly controlled regio- and 1,2-*cis* stereoselective glycosylation. Reaction of 1,2-anhydroglucoside (**85**) as glycosyl donor and unprotected sugar boronic ester derivative (**94**) as glycosyl acceptor predominantly (60–91%) generated the 1→4 linked coupling products (**95**) in exclusive 1,2-*cis* stereoselective fashion also producing a very negligible amount of the corresponding 1→6 linked *cis* glycoside (0–12%). They have successfully used this protocol for glycosylation of PMB protected 1,2-anhydro donor for construction of unprotected natural glycosides and a branched α-linked glucan tetrasaccharide. Based on control experiments and details theoretical studies they proposed that the reaction proceeds through a nine-membered boronic ester (**96**) where the addition of water induces rapid hydrolysis of that ester, inhibiting further activation of the ester for overreaction toward trisaccharide formation resulting in the desired disaccharides.

Catalytic anomeric *O*-alkylation of 1,2-dihydroxyglucoses (**99**) *via* borate complexes with borinic acids, providing 1,2-*cis*-α-glucosides (**101,** up to 95% yield) through predominant activation of axially oriented anomeric oxygens was recently reported by Izumi et al. (2019) (IV in Scheme 9). Optimization showed that both catalysts (**97**) and (**98**) were efficient for the glycosylation in combination with weakly nucleophilic base ^*i*^Pr_2_NEt. Under such mild catalytic condition most of the generally used protecting groups like benzoyl/acetal/silyl easily survived. The steric hindrance at 6-*O*-position dramatically affected the regioselectivity of the reaction. Thus, the reaction of 6-*O*-benzoyl or 6-*O*-unprotected derivatives with 6-*O*-triflate acceptor (**100**) afforded their corresponding 1→6 glycosides in excellent yields. The reaction of bulky 6-*O*-trityl protected dihydoxy donor produced the corresponding 1→6 glycoside in relatively lower yield, as in that case generation of 2→6 glycoside side products increased. In general, these reactions are more efficient with 6-*O*-triflate acceptors. With catalyst (**97**) and triflate at 2, 3, or 4 positions, the desired products were obtained in moderate yields, but changing to catalyst (**98**) dramatically increased the reaction yields. In the absence of catalyst, use of strong base like Cs_2_CO_3_ and 1,2-dichloroethane as the solvent completely changed the selectivity, furnishing *trans*-glycosides exclusively. These results clearly demonstrated the magnitude of the effect of borinic acids in regio- and stereoselective *O*-alkylation even for effective synthesis of model tetrasaccharides.

In late 2020, Tomita et al. (2020) have reported regio- and 1,2-*cis*-α-stereoselective glycosylation reactions of 1,2-anhydroglucose (**85**) with *trans*-1,2-diol sugar acceptor (**102**) using diboron catalyst (V in Scheme 9). This process was used for successful synthesis of several 1,2-*cis* glycosides with significant regioselectivity toward 1→3 glycosides (**103**), the highest 1→3 regioselectivity was achieved for the cyclic protecting group DTBS (di-*tert-*butylsilylene)-based acceptor. Only sharp contrasting regioselectivity was observed when 4,6-OTBS acceptor was used, which resulted in the corresponding 1→2 glycoside (**104**) predominantly. Detail control experiments and extended NMR studies show that the reaction mechanism proceeds through the formation of (**105a**), which activates the anhydro donor (**85**) without any additional additive. Sequential rearrangement of the B-O moiety in (**105b**) affords (**105c**). Finally, a diol exchange between (**103**) and (**105c**) regenerates (**105a**) and provides the 1,2-*cis*-α-glycoside effectively.

### 3.5 Other Methods for 1,2-*Cis* Stereoselective Glycosylation Reactions

**SCHEME 10:**
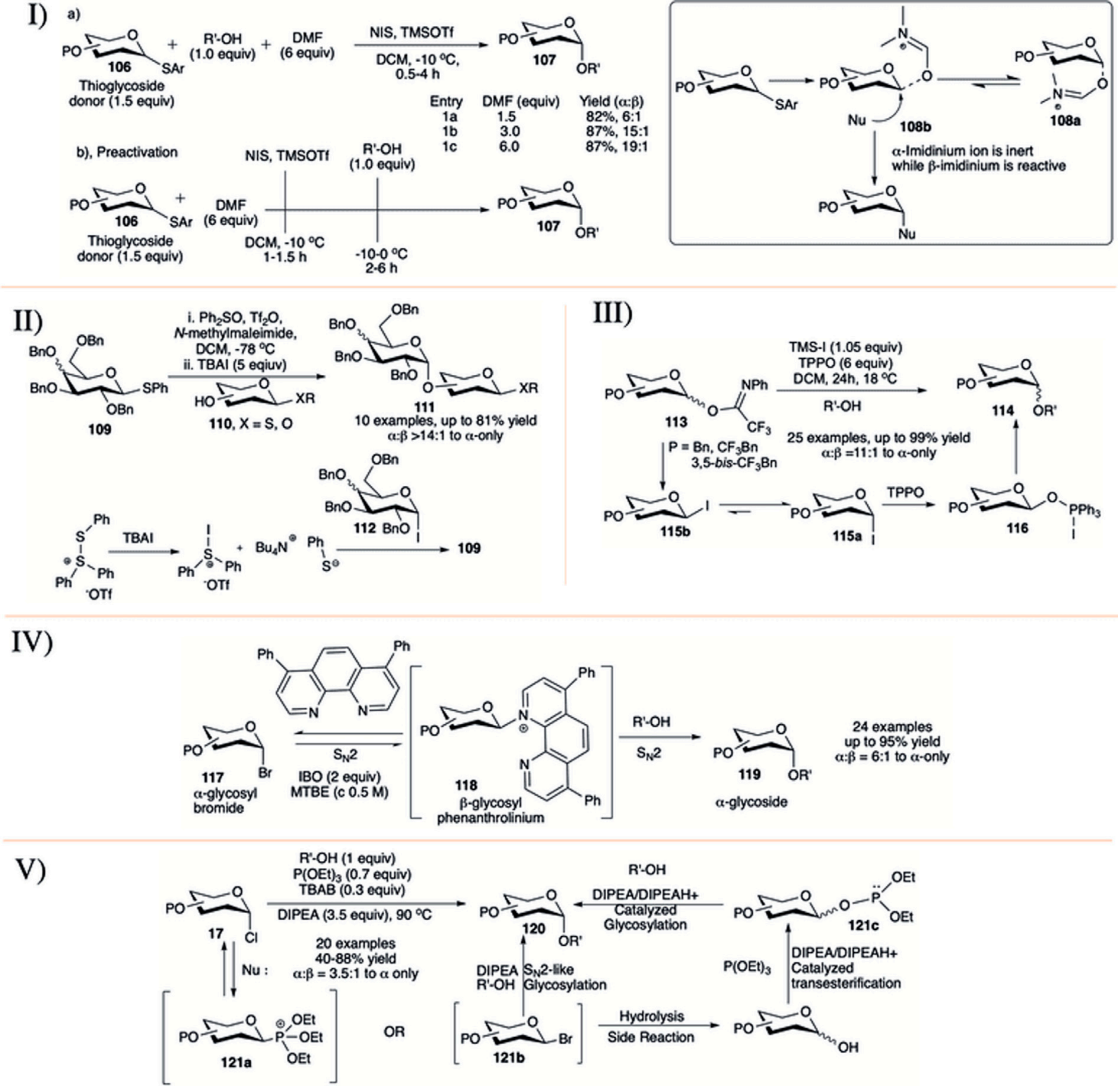
1,2-*cis* glycosylation reactions using I) DMF as co-solvent; II) exogenous substrate TBAI; III) influence of trifluoromethyl benzyl group protection; IV) phenanthroline-catalyst; and V) solvent-free synthesis of α-glycosides from glycosyl chloride donor.

It is well known that ethereal solvents tend to drive the glycosylation toward 1,2-*cis* selective fashion (Eby and Schuerch, 1974; Wulff and Röhle, 1974; Ishiwata et al., 2008). Mong and co-workers took a different direction in studying the reaction solvent effect by using dimethylformamide (DMF) as a co-solvent, a rather uncommon reaction solvent in glycosylation reactions (I in Scheme 10) (Lu et al., 2011; Lin et al., 2012; Liu et al., 2012). This study employed two conceptually different protocols for glycosylation reaction. The first one is a conventional method where in a mixture of O-alkyl protected glycosyl donor (**106),** acceptor and different equivalent of DMF was activated with NIS and TMSOTf to afford disaccharide (**107**) in almost comparable yields and good 1,2-*cis* selectivity (1.5 equivalent DMF, 82%, α:β = 6:1; 3.0 equivalent DMF, 87%, α:β = 15:1; 6.0 equivalent DMF, 87%, α:β = 19:1, entries 1a–1c, I in Scheme 10). The results obtained using these procedures were then applied to the investigation of the effectiveness of the pre-activation-based glycosylation procedure. Accordingly, the thioglycoside donors were reacted with NIS and TMSOTf in the presence of DMF followed by the addition of the glycosyl acceptors giving the disaccharides with almost exclusive 1,2-*cis* selectivity. Encouraged by the 1,2-*cis* stereo-directing effect of DMF, the preactivation protocol was then extended to different sequential and iterative one-pot oligosaccharide synthesis (Liu et al., 2012). In the proposed mechanistic pathway, they suggested that DMF is converting oxocarbenium ion to imidinium ion during thioglycoside activation. α-Imidinium ion (**108a**) is less reactive while β-imidinium ion (**108b**) is reactive generating 1,2-*cis* glycoside predominantly (inset picture, I in Scheme 10), and this was supported by NMR spectra at −20°C (Lin et al., 2012).

In 2013, Bennett and co-workers investigated the activation of thioglycosides with Ph_2_SO/Tf_2_O in the presence of tetrabutylammonium iodide (TBAI) (Chu et al., 2013). The reaction of thioglycoside donor (**109**) and acceptor (**110**) under this condition generated the 1,2-*cis* glycoside (**111**) in moderate to high yields and stereoselectivity (II in Scheme 10). Stereoselectivity of the reaction depends on the quantity of TBAI used and this indicates that the reaction may proceed through a glycosyl iodide intermediate (**112**). Use of excess TBAI can regenerate the starting donor; however, this can be inhibited with the addition of *N*-methylmaleimide as a thiol scavenger. The underlying idea of using TBAI is that the conversion of α-glycosyl triflates into β-glycosyl iodides would favor the formation of α-glycosides. They used this protocol for iterative synthesis of a model trisaccharide in good yield and 1,2-*cis* stereoselectivity.

Use of iodotrimethylsilane (TMS-I) as exogenous iodide source in the presence of triphenylphosphine oxide (TPPO) were further demonstrated by Njeri et al. (2021). Glycosylation reaction of *N*-phenyltrifluroacetimidate donors (**113**) with various primary acceptors resulted their corresponding α-glycosides (**114**) in high yield and stereoselectivity (III in Scheme 10). Stereoselectivity increases with number of trifluoromethyl functionalities on the protecting benzyl groups. Under similar reaction condition, 3,5-*bis*-trifluorobenzyl protected glycosyl donor was more α-selective compared to the corresponding 4-trifluoromethyl benzyl analog. Based on the mechanistic study and recorded literature, the reaction may proceed through initial formation of β-glycosyl iodide (**115b**), which anomerized readily to its corresponding α-iodide (**115a**) followed by the formation of the proposed intermediate (**116**) (not detected by spectroscopic techniques), which then react with glycosyl acceptors to generate the α-glycosides (**114**).

Nguyen’g group reported the commercially available phenanthroline catalyzed stereo-retentive glycosylation of α-glycosyl bromides (IV in Scheme 10) (Yu et al., 2019). They have used 15 mol% of 4,7-diphenyl-1,10- phenanthroline as catalyst, isobutelene oxide (IBO, 2 equivalents) as HBr scavenger in a mixture of glycosyl bromide donor (**117**) and acceptor in *tert*-butylmethyl ether (MTBE) at 50°C for 24 h to generate their corresponding α-glycosides (**119**) in moderate to high yields. Mechanistic studies indicated an initial S_N_2 reaction on α-glycosyl bromides toward formation of β-glycosyl phenanthrolinium ion (**118**) followed by a second S_N_2 attack by the incoming acceptor nucleophile, which resulted in a stereo-retention of the initial glycosidic bond. Reactions with β-glycosyl bromides under different conditions show that the inversion of β-glycosyl bromide to α-glycosyl bromide is much faster than the formation of the glycosidic product, suggesting the progress of the reaction from the α-anomers. They have used this process to proceed in efficient construction of α-glucan polysaccharide and to synthesize glycosides from donor having C2-azido and/or C2-fluoro groups. As a modification toward more efficient synthesis of 2-fluoro glycosides they changed the phenyl ring in the catalyst with piperidine (DeMent et al., 2021).

In the middle of 2020, Traboni et al. (2020) published a process of stereo retention of α-chloro glycosyl donor under air and solvent free condition (V in Scheme 10). They have used the combination of triethyl phosphite (P(OEt)_3_, 0.7 equivalent) as additive, TBAB (0.3 equivalent) as exogenous halide source and DIPEA (3.5 equivalents) as base for the activation of glycosyl-α- chlorides (**17**) to produce the corresponding α-glycosides (**120**) in preeminent yields. They have also used this process to activate glycosyl hemiacetals with additional *in situ* transformation to their corresponding α-glycosyl chlorides. As a plausible mechanism they suggested that the reaction may proceed mainly through the formation of intermediate β-phosphonium ion (**121a**) or -bromide (**121b**) followed by S_N_2-like glycosylation, resulting in the corresponding α-glycosides. Alternatively, these intermediates (**121a**/**121b**) can be hydrolyzed to their corresponding hemiacetal which can generate transient glycosyl phosphite donor (**121c**) and then activated to afford α-glycoside.

### 3.6 α-Stereoselective Glycosylation Reaction for the Synthesis of 2-Deoxyglycosides

The structures of 2-deoxy sugars are diverse, but they all suffer from the absence of stereo directing functionality at 2-position. The anomeric effect tends to dictate a bias toward 2-deoxy-α-glycosides, but the effect is not subtle to be stereospecific during glycosylation reaction.

#### 3.6.1 Thiourea Catalyzed Synthesis of α-Selective 2-Deoxy Glycosides

**SCHEME 11:**
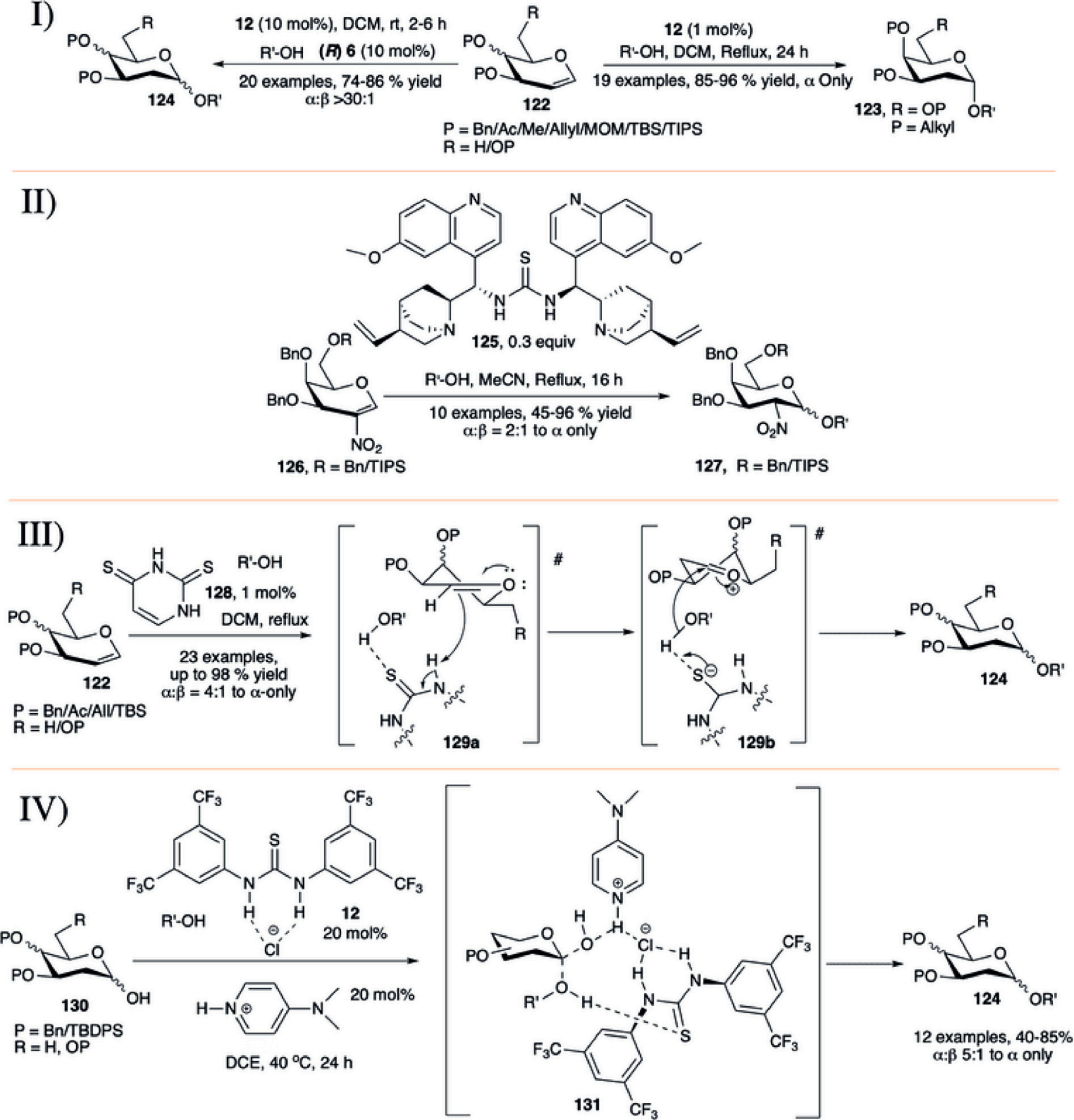
Formation of α-linked 2-deoxyglycosides using I) thiourea-catalyst; II) bifunctional organocatalyst **125**; III) thiouracil-catalyst from glycal donor; and IV) thiourea/DMAP/HCl-catalyst combination from glycosyl hemiacetal donors.

In 2012, Balmond et al. (2012) reported the application of thiourea (**12**) for glycosylation using per-*O*-alkylated d-galactal (**122**) with various carbohydrate acceptors in refluxing DCM toward the α-selective formation of 2-deoxygalactose disaccharides (**123**) in high yields (85–96%, I in Scheme 11). But per-*O*-acetylated d-galactal did not respond under this current reaction condition even after 2 days. They have also evaluated this process for the stepwise and one-pot synthesis of a model trisaccharide in 58% yield with complete stereoselectivity. Then, Palo-Nieto et al. (2017b) used the concept of co-operative acid catalyzed stereoselective glycosylation of versatile protected glycals (**122**) toward their 2-deoxyglycosides (**124**) by a combination of chiral binol-derived phosphoric acid (*R*)-**6** and thiourea substrate (**12**) (I in Scheme 11). Glycosylation reactions utilizing per-*O*-benzylated galactal of different acceptors produced their corresponding 2-deoxygalactopyranosides in high yield and stereoselectivity. Reaction of per-*O*-benzylated d-glucal was relatively lower yielding, and like their earlier report (Balmond et al., 2012) per-*O*-acetylated d-galactal did not respond to the reaction protocol at all.

Working on catalytic stereoselective glycosylation using glycals, Galan’s group introduced 30 mol% of the bifunctional organo-catalyst (**125**) for α-selective glycosylation based on 2-nitrogalactals in refluxing acetonitrile (II in Scheme 11) (Medina et al., 2016). Coupling of 2-nitrogalactal donor (**126**) with different types of acceptors generated their corresponding disaccharides (**127**) in moderate to good yield and stereoselectivity. To showcase the applicability of the current methodology, they used this organocatalytic stereoselective glycosylation using 2-nitrogalactal toward efficient synthesis of mucin-type Core 6 and 7 glycoconjugates.

Bradshaw et al. (2019) introduced thiouracil (**128**) catalyzed α-selective glycosylation of various primary or secondary acceptors using glycals (**122**) in refluxing DCM to generate their corresponding α-selective 2-deoxyglycosides (**124**) in good to excellent yields (III in Scheme 11). Coupling of alkyl protected d-galactal derivatives separately with different acceptors resulted in their corresponding α-2-deoxygalactopyranosides in excellent yields. Reaction of per-*O*-benzyl protected d-glucals or L-rhamnal were relatively lower yielding and poorly stereoselective because of formation of inseparable mixture of Ferrier rearrangement products. This protocol was equally efficient for the construction of 1→1 glycoside with various glycosyl hemiacetal acceptors, and glycosyl sulfonamide in reaction with TsNH_2_. Change in chemical shift for the -NH proton of the catalyst during reaction of the donor with CD_3_OD as acceptor suggested that the reaction proceeds through H-bonding between acceptor and catalyst followed by the attack to the donor forming (**124**) *via* (**129a**) and (**129b**)**.**

Very recently Kancharala *et al.* have reported the influence of anion-binding thiourea (**12**) on 4-dimethylaminopyridine salts toward α-selective synthesis of 2-deoxyglycosides (**124**) from their corresponding hemiacetals (Ghosh et al., 2021). Combination of Schreiner’s thiourea (**12**), DMAP and HCl was used for dehydrative glycosylation of hemiacetals (**130**) in 1,2-dichloroethane solvent at 40°C (IV in Scheme 11). This reaction condition was efficiently used for construction of a varied number of 2-deoxy-α- glycosides. Control experiments, NMR spectroscopic, and theoretical studies suggested that the reaction proceeds through a loose transition state (**131**) where the cationic DMAPH^+^ coordinates with the equatorial isomer of the hemiacetal, and polarized sulfur activates the acceptor leading to the formation of the axially glycosylated products.

#### 3.6.2 Transition Metal-Catalyzed α-Selective Synthesis of 2-Deoxyglycosides

**SCHEME 12:**
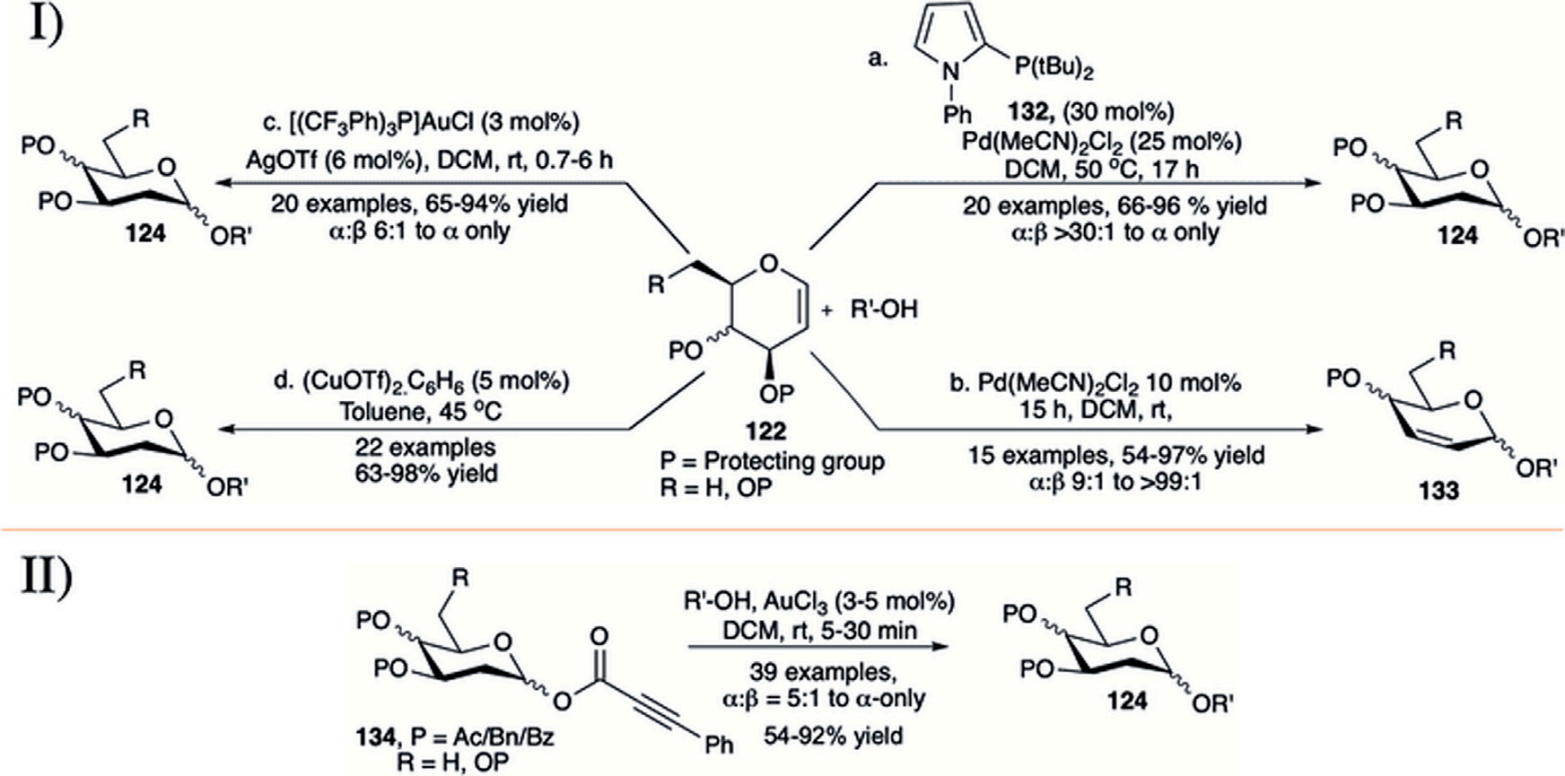
I) Palladium, gold, and copper-catalyzed and II) additive free Au(III)-catalyzed direct α-selective synthesis of 2-deoxyglycoside.

In early 2017, Sau et al. (2017) employed a combination of Pd (II) catalyst and monodentate phosphate ligand (**132**) for direct α-selective glycosylation using glycals (**122**) toward the construction of 2-deoxyglycosides (**124**) (entry a, I in Scheme 12). Reaction protocol was completely silent for reaction with per-*O-*acetylated galactal donor. This technique was found equally efficient for silyl protected d-glucal or L-rhamnal donors. Reaction of per-*O-*allylated d-galactal donor with primary acceptor was relatively low yielding but completely α-selective (68%, α-only). Continuing their investigation on glycosylation of 3-*O*-acylated glycals, Sau *et al.* reported palladium-catalyzed Ferrier type *O-*glycosylation using such donors at room temperature (entry b, I in Scheme 12) (Sau and Galan, 2017). Per-*O-*acetylated d-glucal donor coupled with various primary or secondary acceptors to produce the corresponding 2,3-unsaturated glucoside (**133**) within 3 h in excellent yield and good α-stereoselectivity (entry b, I in Scheme 12). The reactions of per-*O-*acetylated d-galactal gave a mixture of 2,3-unsaturated product (65%) and 2-deoxyglycoside (20%) with almost complete α-stereoselectivity (α:β > 99:1). The 4,6-constrained d-glucal derivative afforded the corresponding product in a lower yield and unchanged stereoselectivity, but surprisingly 4,6-*O*-siloxane protected galactal yielded only the 2-deoxyglycosides in high yield and very high α-selectivity.

Palo-Nieto et al. (2017a) described the use of a combination of [(*p*CF_3_Ph)_3_P]AuCl and AgOTf for direct activation of glycals by the *syn* addition of a proton and oxygen from the nucleophile across the carbon–carbon double bond to yield α-deoxyglycosides (entry c, I in Scheme 12). Glycosylation reactions of different acceptors with d-galactal donor (**122**) furnished the corresponding 2-deoxygalactosides (**124**) in good to high yield and excellent α-selectivity. Partially or completely silyl protected glycal donors were almost equally efficient under current reaction condition, while per-*O-*acetylated d-galactal donor did not produce any desired product. The reaction of 2-deuterated galactal donor yielded 81% of the 2-deoxygalactopyranoside with newly formed bond completely *cis* to each other. They have also used this protocol for synthesis of a model tetrasaccharide in 52% overall yield and maintaining α:β > 30:1 throughout the reaction sequence. Mechanistic studies imply that the reaction proceeds through Au(I)-catalyzed hydro functionalization of the enol ether yielding the 2-deoxyglycoside with high stereoselectivity. In 2020, the same group used 5 mol% of (CuOTf)_2_.C_6_H_6_ in toluene for glycosylation using both armed and disarmed glycals for direct glycosylation forming α-selective 2-deoxyglycosides (**124**) in good to excellent yields (entry d, I in Scheme 12) (Palo-Nieto et al., 2020). Details mechanistic studies and isotope labeling showed that the presence of Cu(I) is essential for efficient catalysis and stereochemical control of the reaction, and the reaction proceeds through dual activation of both the enol ether and the hydroxy nucleophile.

In 2019, Shaw *et al.* used gold (III) chloride for robust activation of 2-deoxy-phenylpropiolate glycosides (d-PPGs **134**) toward α-selective synthesis of 2-deoxyglycosides (**124**) in the absence of any type of additive (II in Scheme 12) (Shaw and Kumar, 2019). Use of 3–5 mol% of AuCl_3_ in DCM at room temperature for 5–30 min was sufficient for the activation of d-PPGs to couple with various acceptors (carbohydrates and non-carbohydrates) producing the corresponding 2-deoxyglycosides (both *O-* and *N-*glycosides) in high to excellent yields and stereoselectivity. This reaction technique was equally efficient for successful activation of 2,6-dideoxyglycosides.

#### 3.6.3 Miscellaneous Methods for α-Selective 2-Deoxyglycoside Synthesis

**SCHEME 13:**
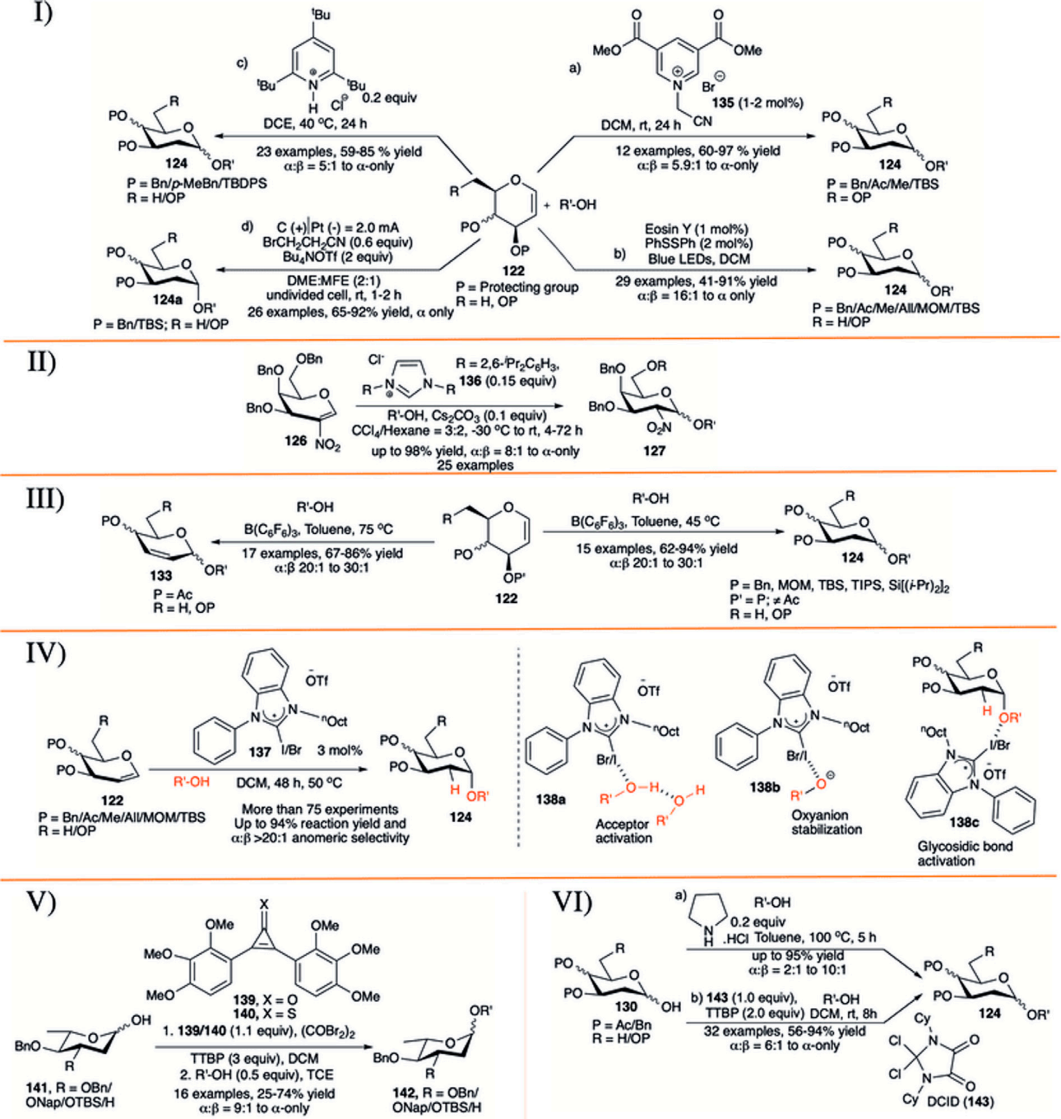
I) a) Pyridinium salt-catalyzed, b) visible-light induced, c) TTBPy-catalyzed, d) electrochemical stereoselective 2-deoxyglcosylation reactions using glycal donors; II) *N*-heterocyclic carbene-catalyzed stereoselective glycosylation reactions using 2-nitroglycals; III) substrate-controlled α-selective glycosylation reactions of glycals; IV) halogen bond organo-catalyzed 2-deoxyglycosylation reactions; V) reagent-controlled α-selective glycosylation reactions of 2,6-dideoxy sugars; and VI) a) secondary amine-catalyzed, b) dichloroimidazolidinedione (DCID)-promoted stereoselective glycosylation reactions of 2-deoxy hemiacetals.

In 2015, Das et al. (2015) reported electron-deficient pyridinium salt (**135**) as an efficient catalyst (1–2 mol% catalyst loading) for activation of glycals (**122**) toward the formation of their corresponding disaccharides (**124**) in excellent yield and α−stereoselectivity (entry a, I in Scheme 13). The general reaction-yields are very high with alkyl protecting groups (Bn/Me/TBS) on glycal donor, but glycals with acetate protection did not respond at all. The anomeric selectivity of the glycosylation reactions was always very high (α:β = 33:1 to α-only), with only exception for the reaction with methanol (α:β = 5.9:1). In 2018, Zhao *et al.* reported visible-light induced photoacid catalyzed α-selective synthesis of 2-deoxyglycosides (**124**) from glycals (**122**) in the presence of 2 mol% of PhSSPh as additive (entry b, I in Scheme 13) (Zhao and Wang, 2018). This reaction sequence was very successful with almost every types of glycal donors, irrespective of protection profile on it and affording the corresponding 2-deoxyglycosides (**124**) in moderate to excellent yields and α-stereoselectivity. More importantly, this protocol efficiently overcomes the major drawbacks of previously reported methods (Balmond et al., 2012; Palo-Nieto et al., 2017a, 2017b; Sau et al., 2017) for direct synthesis of 2-deoxyglycosides from glycals, avoiding competitive Ferrier rearrangement reaction of C-3 O-acetyl protected glycals.

Ghosh et al. (2019) further demonstrated that the protonated 2,4,6-tri-*tert*-butylpyridine salt serves as bulky and strained catalysts for efficient and highly stereoselective glycosylation reactions of various glycals (entry c, I in Scheme 13). They have explored the efficacy of the catalyst for a variety of differentially protected glycal donors (**122**) and various acceptors generating their corresponding 2-deoxyglycoside **124**, and also extended its applicability in gram-scale synthesizing norbornene–glycoside. The mechanistic study *via* NMR monitoring of the reaction course suggests that the reaction involves an interesting single-hydrogen bond mediated protonation of glycals. The counter ion also plays crucial role in the outcome of the reaction; thus, changing chloride ion with ^(-)^B [C_6_H_3_(CF_3_)_2_]_4_ ion directs the reaction toward Ferrier rearrangement product. Liu et al. (2020) reported a novel approach for stereoselective electro-2-deoxyglcosylation from glycals (entry d, I in Scheme 13). The electrochemical glycosylation reactions with glycals (**122**) of various acceptors were performed in an undivided cell filled with 0.6 equivalent of BrCH_2_CH_2_CN, 2 equivalents of Bu_4_NOTf in 2:1 mixture of dimethoxy ethane (DME):methyl nonafluorobutyl ether (MFE) under constant current gradient of 2.0 mA for 1–2 h to generate their corresponding α-2-deoxyglycosides (**124a**) in very good yields. This method features excellent scope and functional-group tolerance, applicability of stereoselectivity in modifications of wide range of natural products and drugs, synthesis of glycosylated podophyllotoxin, and a one-pot trisaccharide *via* iterative electro-glycosylation reactions.

In 2017, Liu et al. (2017) introduced *N*-heterocyclic carbene (**136**) for stereoselective glycosylation of 2-nitroglycals in a 3:2 mixture of CCl_4_:hexane as solvent. 2-nitrogalactal donor (**126**) coupled with various acceptors producing their corresponding glycoside (**127**) in high to excellent yield and 1,2-*cis* selectivity (II in Scheme 13). They have also shown the applicability of this reaction in the gram scale.

Sau et al. (2019) described B(C_6_F_6_)_3_ catalyzed temperature-dependent substrate-controlled α-selective glycosylation using glycal donors (III in Scheme 13). Reaction of *O-*acetylated glycals with various acceptors at 75°C produced their 2,3-unsaturated glycosides (**133**) in good yield and stereoselectivity. The reaction of glycal donors (**122**), without *O-*acetyl protection adjacent to double bond produced 2-deoxyglycosides (**124**) in good yield and stereoselectivity at relatively lower temperature (45°C).

Xu et al. (2020) reported noncovalent halogen bonding (XB) catalyzed α-stereoselective 2-deoxyglycosylation based on d-glycals (IV in Scheme 13). This report described the use of noncovalent XB catalyst (**137**) for the glycosylation using a variety of d-glycals (**122**) of numerous different acceptors which resulted in the corresponding 2-deoxyglycosides (**124**) in excellent yields and α-stereoselectivity. Kinetic investigations, control experiments, and NMR studies reveal an auto inductive sigmoidal kinetic profile, supporting an *in situ* amplification of XB-dependent active kinetic species. The proposed mechanism suggests that the reaction starts with acceptor activation (**138a**) by the XB catalyst, which proceeds further to exist as a solvent separated ion pair to give oxyanion stability (**138b**). This (**138b**) can subsequently protonate C2 of glycal (**122**) and transfer acceptor *via* glycosidic bond activation (**138c**). Deuterium labeling from acceptor confirms the transfer of proton to C2 of glycal from the acceptor moiety. As a benchmark of this protocol, they have compared and entrenched the superiority of this protocol with some of the previously reported thiourea catalyst (**12**) (Balmond et al., 2012) and (**128**) (Bradshaw et al., 2019) used for similar purposes.

Apart from use of glycal for α-selective 2-deoxyglycoside synthesis, 2-deoxyhemiacetals were also used for a couple of times. Continuing their work on reagent controlled 2-deoxy α-selective glycosylation, Bennett’s group reported that combination of 2,3-bis(2,3,4-trimethoxyphenyl)cyclopropenone (**139**) or 2,3-bis(2,3,4-trimethoxyphenyl)cyclopropene-1-thione (**140**) with oxalyl bromide in the presence of additive TTBP, endorses the glycosylation reaction between 2,6-dideoxy-sugar hemiacetals (**141**) and glycosyl acceptors in good yield and high α-selectivity (V in Scheme 13) (Nogueira et al., 2016). Reaction of 3-*O*-benzyl/napthyl/TBS 2,6-dideoxy or 2,3,6-tri-deoxyhemiacetal donor (**141**) separately with primary acceptors using promoter (**139**) furnished the corresponding disaccharides **142** in high yield and good anomeric selectivity. In the case of secondary acceptors, they found that the combination of oxalyl bromide with 1-thione substrate (**140**) was more efficient toward fruitful glycosylation reaction, whereas this combination was not so effective for reaction with primary acceptor.

In 2018, Ghosh et al. (2018) explored the efficiency of simple pyrrolidine hydrochloride as an organocatalytic activator for stereoselective glycosylation of furanose and pyranose hemiacetals (**130**) to synthesize 2-deoxyglycoside (**124**) (entry a, VI in Scheme 13). Interestingly, when 2-deoxy-glucosyl hemiacetal was allowed to participate under the present reaction condition in the absence of any additional acceptor, it dimerizes generating α→α-trehalose analog in 75% yield. They showed that the current robust glycosylation protocol is equally efficient for glycosylation of sterically hindered acceptors as well as in the construction of furanosides. Very recently Javed et al. (2022) reported commercially available geminal dichloroimidazolidinedione (DCID, **143**) as a source of 4,5-dioxo-imidazolium cation for α-selective glycosylation of 2-deoxy and 2,6-diexoy glycosyl hemiacetals. Reaction of various 2-deoxy and 2,6-diexoy glycosyl hemiacetals (**130**) with different acceptors in the presence of 1 equivalent DCID and 2 equivalent TTBP in DCM solvent produced their corresponding glycosides (**124**) in moderate to high yield and stereoselectivity (entry b, VI in Scheme 13). Reactions were equally efficient with both sugar and non-sugar acceptors, including amino acids, but reaction yield with secondary sugar acceptor was relatively lower.

## 4 Selectivity Tuning During Glycosylation Reactions

**SCHEME 14:**
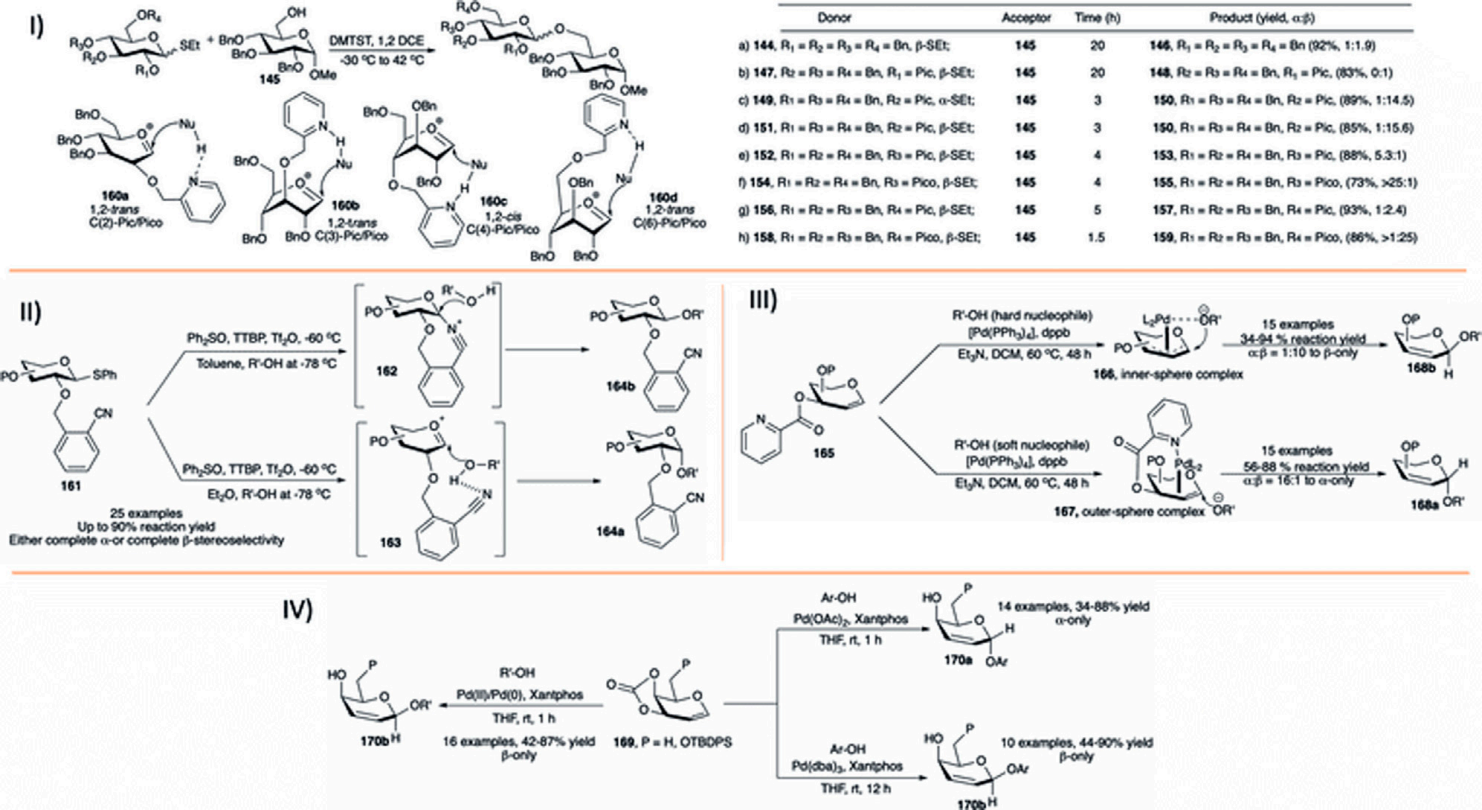
I) Stereoselectivity tuning with change in picolinyl (Pic)/picoloyl (Pico) position; II) solvent-directing stereo-modulation using 2-cyanobenzyl ether protected glycosides; III) stereoselectivity modulation using inner-sphere, outer-sphere complex formation during glycosylation reactions; and IV) Pd (0)/Pd(II)-catalyst-controlled stereoselective *O-*glycosylation reaction of 3,4-carbonate glycal donors.

In a detailed report, Demchenko *et al.* studied the effect of adjacent and remote picolinyl and picoloyl substituent on the stereoselectivity of chemical glycosylation (Yasomanee and Demchenko, 2012). They studied the effect of the picolinyl group separately at C-2, C-3, C-4, and C-6 as well as the effect of the picoloyl group and donor concentration on glycosylation reaction. Glycosylation with per-*O*-benzylated thioglycoside donor (**144**) of primary sugar acceptor (**145**) using dimethyl (methylthio)sulfoniumtriflate (DMTST), 1,2-dichloroethane, at −30–42 °C yielded their corresponding disaccharide (**146**) in a non-stereoselective fashion (92%, α:β = 1:1.9, entry a, I in Scheme 14). Coupling of the corresponding 2-*O*-picolinyl donor (**147**) with the same acceptor (**145**) provided disaccharide (**148**) with anticipated exclusive 1,2-*trans* stereoselectivity in 83% yield (entry b, I in Scheme 14). Glycosylation with both α- and β-anomers of ethyl 2,4,6-tri-*O*-benzyl-3-*O*-picolinyl-1-thio-d-glucopyranosides (**149** and **151**) of acceptor (**145**) generated their corresponding disaccharide (**150**) in almost similar yields and stereoselectivity (89%, α:β = 1:14.5 and 85%, α:β = 1:15.6, respectively, entries c and d, I in Scheme 14). Use of picoloyl substituent at the C-4 position instead of picolinyl protection significantly enhances 1,2-*cis* stereoselectivity. While reactions of glycosyl acceptor (**145**) with 4-*O*-picolinyl donor (**152**) gave unexceptional stereoselectivity of disaccharide (**153**) (α:β = 5.3:1, entry e, I in Scheme 14), 4-*O*-picoloyl donor (**154**) led to disaccharide (**155**) with almost complete 1,2-*cis* stereoselectivity (73%, α:β > 25:1, entry f, I in Scheme 14). Conversely, glycosylation using 6-*O*-picolinyl donor (**156**) (substituent projecting from above the ring) produced disaccharide (**157**) with moderate β-stereoselectivity (α:β = 1:2.4, 93%, entry g, I in Scheme 14). Interestingly, the 6-*O*-picoloyl donor (**158**) showed excellent 1,2-*trans* stereoselectivity, and the resulting disaccharide (**159**) (96%, α:β > 1:25, entry h, I in Scheme 14) was obtained in much lower reaction time. Thus, picolinyl/picoloyl protection on C-2, C-3, and C-6 position leads to 1,2-*trans* selective glycosylation whereas that protection at C-4 directs the glycosylation reaction toward 1,2-*cis* selectivity. The reason behind this tuning of stereoselectivity with the change of position of picolinyl/picoloyl protection is due to the fact of H-bonded aglycon delivery (HAD) (**160a**-**160d**, I in Scheme 14). In support of this hydrogen-bonding concept they ran multiple experiments; in one such reaction 10-fold dilution in the donor concentration increases stereoselectivity 3- to 4-folds, and the reaction completed within much shorter time. Stereoselectivity decreases with the addition of DMSO, as H-bonding is disturbed by the addition of it. The reaction of donor where N-H of picolinyl was replaced with *N*-TMS is non-stereoselective. Further decrease in stereoselectivity was observed when *m*-or *p*-picolinyl/picoloyl derived donors were used for glycosylation.

In 2012, Liu *et al.* studied interesting solvent-assisted dual-directing auxiliary of 2-cyanobenzyl ether for a highly stereoselective glycosylation reaction (Le Mai Hoang and Liu, 2014). Pre-activation of C2-2-cyanobenzyl ether protected glycosyl donor (**161**) with Ph_2_SO, TTBP, and Tf_2_O at −60°C followed by addition of acceptor at −78°C generated their corresponding glycosides in excellent yield and stereoselectivity, depending on the reaction solvent used (II in Scheme 14). Glycosylation reaction in toluene proceeds through the transition state (**162**) (supported by NMR spectra at −78°C), resulting in β-glycosides (**164b**), exclusively. For reaction with Et_2_O solvent, the reaction proceeds through H-bonded intermediate (**163**) (supported by the trapping experiment and H-bonding disruption study) exclusively produced their corresponding α-glycosides (**164a**) in excellent yields. This solvent directed the 2-cyanobenzyl ether mediated stereoselectivity modulation process was equally efficient for glycosylation reaction of TMSOTf-promoted OTCA donors. The reaction protocol was unresponsive with glycosyl acceptors carrying the -OH group at 4-position. Continuing their research on stereo-diverse glycosylation techniques, Liu *et al.* used the effect of remote 3-*O-*picoloyl group on glycal donors for reversing stereoselectivity of Pd-catalyzed *O-*glycosylation through an inner-sphere or outer-sphere intermediates (III in Scheme 14) (Xiang et al., 2015). Reaction of glycal donors (**165**) bearing remote 3-*O-*picoloyl group with various hard acceptors (like aliphatic alcohols or carbohydrate acceptors) *via* the formation of an inner-sphere complex (**166**), resulting in their corresponding glycosides (**168b**) in good yield and β-stereoselectivity. Similar reaction with the soft nucleophiles like phenols, formed outer-sphere complex (**167**) generating their corresponding glycosides (**168a**) in excellent yield and α-stereoselectivity. The lower reaction yield was attributed to the steric hindrance of the bulky tertiary acceptor, 1-adamentanol or 2,6-dimethyl phenol.

In 2017, the same group reported Pd (0)/Pd(II) catalyst-controlled stereoselective glycosylation of 3,4-carbonate glycal donors (**169**) in moderate to high yield and exclusive α- or β- stereoselectivity (IV in Scheme 14) (Yao et al., 2017). With Pd(II) catalyst, hard nucleophiles like aliphatic alcohols or sugar acceptors gave β-glycosides (**170b**), and α-glycosides (**170a**) were obtained from soft nucleophiles like phenols (IV in Scheme 14). On the contrast Pd (0) catalyst gave β-glycosides (**170b**) with both soft (phenols) and hard nucleophiles (sugar acceptors) due to H-bonded coordination of the catalyst with donor from β-face and subsequent H-bond-mediated aglycone delivery (IV in Scheme 14). Relatively low reaction yield was observed for reaction with sterically hindered acceptor like 2,6-dimenthyl phenol (44 and 34% for Pd (0) and Pd(II) catalyzed reaction, respectively) or 4-OH of glucoside acceptor (42%).

## 5 Conclusion

In past decades, glycosylation research has been oriented toward the utilization of catalytic activators or activator- systems for efficient glycosylation reactions in terms of yield and anomeric selectivity. In the current review, we have tried to highlight the recent trend on efficient glycosylation methods reported during the last decade for stereoselective synthesis of α- or β-glycosides. At the end we have included a table (Table 1) mentioning the merits and demerits of these glycosylation methodologies. It is hoped that this overview will help synthetic carbohydrate researchers to choose a target oligosaccharide synthesis at a glimpse from the table suitable glycosylation methods out of the embodied ones therein.

**TABLE 1:** Schematic presentation of the developed methods with achievements and shortfalls.

Selectivity	Glycosyl donor	Mode of glycosylation	Catalyst	Achievement	Shortfall	Reference
β-Selective	Glycosyl–OTCA	Acid–base catalysis	PhBF_2_ or Ph_2_BF	Excellent stereoselectivity and general reaction yields	Low yield and less selective for sterically hindered secondary acceptors	Kumar et al. (2011)
			PhSiF_3_			Kumar et al. (2012)
			AuCl_3_ or AuCl			Peng and Schmidt (2015)
		Co-operative catalysis	(*R*)-**6** or (*S*)-**6** (Chiral phosphate)	Good stereoselectivity with (*S*)-**6**	Highly protecting group-dependent and no detail study on donor protection profile	Cox et al. (2010)
				**12** (thiourea) and **13** (phosphate)	Good reaction yields and stereoselectivity	Not selective with sugar acceptors	Geng et al. (2013)
					**15** (Salen-Cobalt catalyst)	Bench-stable promoter and room temperature reaction	Though surprising α-selectivity for some reaction but inconsistent anomeric selectivity	Medina et al. (2015)
	Glycosyl chloride		(*R,R*)-**16,** (Macrocyclic thiourea)	Excellent stereoselectivity and reaction yields	Low yield and less selective for sterically hindered secondary acceptors	Park et al. (2017)
	Glycosyl phosphate		**18**, (Macrocyclic bis-thiourea)		Carbon-centered nucleophiles were unreactive	Levi et al. (2019)
	Glycosyl–OTCA	Transition metal	FeCl_3_	Environmentally benign catalyst	Low temperature reaction condition with hygroscopic catalyst	Mukherjee et al. (2016)
	Thioglycoside	Reagent controlled	**25**	Operationally simple, consistent solvent effect	Some sugar acceptors showed high yield with low selectivity or low yield with high stereoselectivity	Chu et al. (2015)
	Mesylate	Borinic ester	Oxaboraanthracene-derived borinic acid	Excellent stereo- and regio-selectivity	Low yield and stereoselectivity with 4,6-*O*-benzylidene donors	D'Angelo and Taylor (2016)
	Thioglycoside or PTFAI	2,3-*O*-Xylene protection	NIS/TMSOTf Or BF_3_.Et_2_O	High yield and good stereoselectivity	Electron withdrawing group at 4 and/or 6 positions decrease stereoselectivity	Yagami et al. (2017)
	Imidate	-OFox	Lewis’s acid	Excellent yield and stereoselectivity	Not selective with sterically hindered acceptors	Nigudkar et al. (2017)
			BrØnsted acid			H_3_Pypy	Nielsen et al. (2022)
		Thioglycoside	2-Cyano ethyl protection			NIS/TMSOTf	Molla et al. (2020)
			One or more free hydroxyl of donor		Bu_2_B-OTf, Ph_2_SO, and Tf_2_O	Stereoselectivity reduces in presence of acetyl group adjacent to free -OH and for 6-OH free donor	Le Mai Hoang et al. (2017)
		Glycosyl–OTCA	2-F-2-deoxyglycoside	TMSOTf	Moderate yield but excellent stereoselectivity	Low yield and stereoselectivity with sterically hindered secondary acceptor	Durantie et al. (2012)
		Glycosyl hemiacetals	*In situ* generation of glycosyl sulfonates	KHMDS, *N*-tosyl 4-nitroimidazole	Efficient for both *O*- and *S*-nucleophile	Low yield with acetonide protected acceptor	Issa et al. (2013)
		Glycosyl-OABz	H-bond mediated	Ph_3_PAuBAr_4_, **49** (Phosphine oxide)	Excellent yield and stereoselectivity	Low selectivity for less sterically hindered and electron rich primary acceptor	Zeng et al. (2019)
			Glycosyl chloride	Organoboronic acid	Boronic acid, Ag_2_O	Excellent yield, regio- and stereoselectivity	No reaction with 4-OH position of acceptor	Beale et al. (2014)
α-Selective			Co-operative catalysis	**12**, (Thiourea)	High yield and stereoselectivity in most cases	Concentration-dependent and very low yield with glucose 4-OH acceptor	Hashimoto et al. (2016)
		2,6-lactone-OTCA		**57** (Urea)		Low yield for glucosyl chloride donor	Sun et al. (2016)
	Diphenyl phosphate	**18/59** (Macrocyclic thiourea)		Low selectivity with simple alkyl protected donor		Li et al. (2020)
	Glycal	**12** (Thiourea)			Limited study with D-galactal and not effective with *O*-acetyl protection next to the double bond	Balmond et al. (2012)
		**125** (Thiourea)			Low yield with acceptors bearing electron withdrawing protection and low selectivity with non-sugar alcohols	Medina et al. (2016)
		***R*-6** (Chiral phosphate) **12** (Thiourea)			Low yield for glucal donor and no reaction with acetyl protection next to the double bond	Palo-Nieto et al. (2017b)
			**128** (Thiouracil)		Low yield with secondary acceptor and no reaction with acetyl protection next to the double bond	Bradshaw et al. (2019)
		Transition metal	**132** (Phosphate), Pd (MeCN)_2_Cl_2_			Sau et al. (2017)
			Au(I)/AgOTf			Palo-Nieto et al. (2017a)
			Cu(OTf)_2_.C_6_H_6_			Palo-Nieto et al. (2020)
			Pd (MeCN)_2_Cl_2_		Low yield for acetylated galactal and 4,6-constrained glucal	Sau and Galan (2017)
	Glycosyl–OTCA		Ni(4-F-PhCN)_4_(OTf)_2_		Not selective with sterically hindered acceptors	Mensah and Nguyen (2009)
						Mensah et al. (2010)
	PPGs		AuCl_3_		Low yield with 3-OH free acceptor	Shaw and Kumar (2019)
	PTFAIs	Leveraging CF_3_Bn protection	TMS-I, TPPO		Low yield with 2-OH free acceptor, not a general approach	Njeri et al. (2021)
	1,2-anhydro sugar	Boronic ester	*p*-F-PhB(OH)_2_		Low yield with hindered secondary acceptors	Nakagawa et al. (2015)
			*p*-NO_2_-PhB(OH)_2_			Nishi et al. (2018)
			[B(OH)_2_]_2_		Regioselectivity varies with donor protection profile	Tomita et al. (2020)
	1,2-dihydroxy		**97**/**98**		Steric hindrance at 6-*O*-position dramatically affects the yield and regioselectivity of the reaction	Izumi et al. (2019)
	Thioglycoside	DMF modified	NIS/TMSOTf		Low yield with hindered secondary acceptors	Lu et al. (2011)
						Lin et al. (2012)
						Liu et al. (2012)
		TBAI modifies	Ph_2_SO, Tf_2_O			Chu et al. (2013)
	Glycosyl bromide	Double inversion	1,10-Phenanthroline		General low yield with secondary acceptors	Yu et al. (2019)
						DeMent et al. (2021)
	Glycosyl chloride		P(OEt)_3_, TBAB, DIPEA		Low yield with axial 4-OH galactosyl acceptors	Traboni et al. (2020)
	Glycosyl hemiacetal	Co-operative catalysis	**12,** DMAP.HCl		No study with secondary acceptor	Ghosh et al. (2019)
		Secondary amine	Pyrrolidine.HCl			Ghosh et al. (2018)
		Catalyst controlled	DCID, TTBP		Low yield with secondary sugar acceptors	Javed et al. (2022)
			**139/140** (COBr)_2_		Low yield with hindered secondary acceptors	Nogueira et al. (2016)
	Glycal		**135** (Pyridinium salt)		No reaction with acetylated glycals	Das et al. (2015)
			B(C_6_F_6_)_3_		Reaction course is substrate and temperature dependent	Sau et al. (2019)
			**136** (*N*-heterocyclic carbene)		No study with hindered acceptor	Liu et al. (2017)
			**137** (Halogen bond organo catalyst)		Low yield with simple alkyl protected glycals and no study on Ferrier rearrangement.	Xu et al. (2020)
			2,4,6-tri-*tert*-butylpyridine salt		Low yield with hindered secondary acceptors	Ghosh et al. (2019)
		Visible light induced	Blue LED, Eosin Y			Zhao and Wang (2018)
		Electrochemical	-		Large excess of donor required, and Ferrier rearrangement was not studied	Liu et al. (2020)
Selectivity tuning	Thioglycoside	Remote picolinyl (Pic)/picoloyl (Pico) protection	DMTST	General high yield and stereoselectivity on either direction	Not good for α-galactoside synthesis.	Yasomanee and Demchenko (2012)
	Thioglycoside and/or glycosyl–OTCA	2-Cyanobenzyl ether protection at C-2 position and solvent effect	Ph_2_SO, TTBP, Tf_2_O and/or TMSOTf		No reaction with 4-OH glycosyl acceptor	Le Mai Hoang and Liu (2014)
	3-*O-*picoloyl glycal	Inner-sphere, outer-sphere complex formation	Pd(PPh_3_)_4_		Low yield with sterically hindered phenol and 4-OH of glucoside acceptor	Xiang et al. (2015)
	3,4-carbonate glycols	Pd (0)/Pd(II) catalyst controlled	Pd(OAc)_2_ or Pd (dba)_3_			Yao et al. (2017)

Although like natural glycosylation an ideal chemical glycosylation reaction should be based on unprotected glycosyl donor–acceptor partners in aqueous medium but development of highly regio- and stereoselective chemical glycosylation using native sugar-monomers in aqueous medium seems practically impossible because of the presence of so many free nucleophilic OH groups, and water molecule is a better competing nucleophile/glycosyl acceptor. Alternately, with the existing knowledge-bank research may also be directed toward developing size-controlled stable micellar medium as “micro-reactor” or “nano-reactor” (La Sorella et al., 2015; Paprocki et al., 2018) for organized housing of protected glycosyl donor–acceptor pairs as well as suitable catalytic activator/additives in the confined space of the “reactor” to carry out such reactions in water at a faster rate with high stereo-selectivity.

Recently, after resolving some of the earlier bottlenecks and utilizing AGA-technique, synthesis of a polysaccharide 151-mer has been achieved efficiently by Seeberger’s group (Joseph et al., 2020). Thus, let us hope that the other existing hurdles toward a unified glycosylation method will also be resolved in the near future. Only ongoing efforts can answer the question of whether a universal glycosylation can be implemented. Can this become like peptide and DNA synthesizers where a fully automated universal oligosaccharide/polysaccharide synthesizer be developed? Future work will make this goal more accessible.
